# A comprehensive review of cell transplantation and platelet‐rich plasma therapy for the treatment of disc degeneration‐related back and neck pain: A systematic evidence‐based analysis

**DOI:** 10.1002/jsp2.1348

**Published:** 2024-06-24

**Authors:** Jordy Schol, Shota Tamagawa, Tibo Nico Emmie Volleman, Muneaki Ishijima, Daisuke Sakai

**Affiliations:** ^1^ Department of Orthopedic Surgery Tokai University School of Medicine Isehara Japan; ^2^ Tokai University Center of Regenerative Medicine Isehara Japan; ^3^ Department of Medicine for Orthopaedics and Motor Organ Juntendo University Graduate School of Medicine Tokyo Japan

**Keywords:** back pain, cell therapy, clinical significance, clinical trial, discogenic pain, neck pain, platelet‐rich plasma, regenerative medicine, spine, systematic review

## Abstract

Low back pain (LBP) and neck pain predominate as the primary causes of disability. Cell‐ and platelet‐rich plasma (PRP) products are potential therapies with clinical trials and reviews promoting their efficacy. Nonetheless, they frequently disregard the clinical significance of reported improvements. In this systematic review, the effectuated improvements in pain, disability, quality of life (QoL), and radiographic images are comprehensively described and scored on their clinical significance. An electronic database literature search was conducted on July 2023 for in‐human assessment of cell or PRP products to alleviate discogenic pain. Papers were screened on quantitative pain, disability, QoL, radiographic improvements, and safety outcomes. Risk of bias was assessed through MINORS and Cochrane Source of Bias tools. Reported outcomes were obtained, calculated, and assessed to meet minimal clinically important difference (MCID) standards. From 7623 screened papers, a total of 80 articles met the eligibility criteria, presenting 68 specific studies. These presented at least 1974 treated patients. Overall, cell/PRP injections could alleviate pain and disability, resulting in MCID for pain and disability in up to a 2‐year follow‐up, similar to those observed in patients undergoing spinal fusion. Included trials predominantly presented high levels of bias, involved heterogeneous study designs, and only a minimal number of randomized controlled trials. Nonetheless, a clear clinically significant impact was observed for cell‐ and PRP‐treated cohorts with overall good safety profiles. These results highlight a strong therapeutic potential but also underline the need for future cost‐effectiveness assessments to determine the benefits of cell/PRP treatments.

## Graphical abstract

This comprehensive review of 80 human studies reveals that cell‐ and platelet‐rich plasma (PRP) therapies offer significant relief for low back and neck pain, achieving important clinical benchmarks for pain reduction and disability. Despite varying trial quality, results are promising with safety on par with spinal surgeries, highlighting the strong potential of these innovative treatments in managing chronic pain.

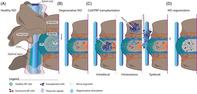

**Abbreviations**
ADC: apparent diffusion coefficient
AF: annulus fibrosis
BMA: bone marrow aspirate
BMC: bone marrow concentrate
LBP: low back pain
HA: hyaluronic acid
IVD: intervertebral disc
MCID: minimal clinically important difference
MINORS: methodological index for nonrandomized studies
MRI: magnetic resonance imaging
MSC: mesenchymal stromal cell
NP: nucleus pulposus
NPS: numerical pain scales
NRS: numerical rating scale
ODI: Oswestry disability index
ORS: Orthopedic Research Society
PRP: platelet‐rich plasma
QoL: quality of life
SAE: serious adverse events
SF: short form
VAS: visual analogue scale

## 1 BACKGROUND

Cellular therapeutics are a rapidly evolving research field, and the market is projected to expand significantly.^1^, ^2^, ^3^ It entails the introduction of de novo cells that replace, complement, or provide a new capacity to the endemic population in the body. The transplanted cells can thereby integrate in situ and support tissue production or general homeostasis or, alternatively, may involve a temporal presence targeted at directing, activating, or attracting native cells.^4^ As such, the application of (stem) cells as a means of therapy poses a potential paradigm shift in medicine. The most established form of cell therapy involves the transplantation of bone marrow aspirates in treated leukemia patients to repopulate their depleted bone marrow. Since then, new techniques and ideas have led to the development of cellular transplantation products for a range of applications, for example, oncogenic CAR‐T, receding gum allografts, and cord blood products.^5^, ^6^ The predominant applications of cell therapy are focused on hematological and tumorous diseases, followed by their usage in the motor system, central nervous system, autoimmune or graft versus host diseases.^7^ Here, transplants are proposed to effectuate their therapeutic effect either by limiting pathological inflammatory or immunogenic conditions or by supporting/stimulating the regeneration of diseased tissues. These aspects provide the prospect for the repair of tissues with limited inherit regenerative capacity, for example, cartilage and nervous system tissues, that were previously considered unmendable and thus have garnered significant attention.

**FIGURE 1:**
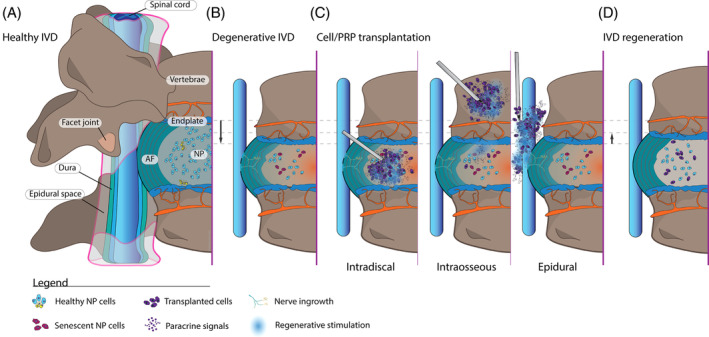
Illustration presenting the general structure of the intervertebral disc (IVD) and the impact of disc degeneration and cell therapy‐derived regeneration. (A) A healthy IVD as an entire unit, including the nucleus pulposus (NP), annulus fibrosis (AF), and vertebra with facet‐joints. (B) Progression of disc degeneration illustrated by loss of matrix quality, AF‐organization, loss of endplate permeability, and NP cell viability and activity, which in turn may promote nerve ingrowth. (C) Transplantation of de novo cells or PRP products into the disc, vertebrae, or epidural space, may support (D) regeneration of the IVD as hallmarked by improvement in cell quantity and quality, tissue organization, nerve ingrowth and sensitization, biomechanical features, and disc features such as height and hydration. AF, anulus fibrosus; IVD, intervertebral disc; NP, nucleus pulposus; PRP, platelet‐rich plasma.

Low back pain (LBP) and neck pain form the primary causes of disability worldwide and present an exceptionally high economic burden on societies.^8^ The 2021 global burden of disease study has once again highlighted the severe impact of LBP,^8^ particularly among the workforce population.^9^ Although LBP and neck pain are recognized as multifactorial diseases,^10^, ^11^, ^12^, ^13^ a plurality of cases are found to be associated with the degeneration of the intervertebral disc (IVD)^12^ (Figure 1a,b). Here, the fibrocartilage IVD structure progressively loses its specialized organization involving a central hydrophilic nucleus pulposus (NP) and a meticulously organized lamellar annulus fibrosus (AF).^14^ Due to severely restricted vascularization, limited to the regions bordering the vertebrae (i.e., the endplates) and the peripheries of the outer AF, the IVD is burdened with a limited self‐repair capability.^15^ In part, the avascular nature results in limited gas, nutrient, and cell replenishment, and the overall cell density in the IVD is low compared to other tissue structures.^16^, ^17^, ^18^ Moreover, the quality of cells shows a rapid decline, with a loss of progenitor cell types and a switch from active proteoglycan‐producing cells to fibrotic and senescent cell types.^19^, ^20^, ^21^ This complex and progressive pathway of structural destruction is complemented by the secretion of inflammatory factors that may promote the influx of immunogenic cells, worsening the pathology.^22^, ^23^, ^24^, ^25^

Despite increasing efforts, treatment strategies for discogenic LBP remain primarily palliative or are centered on full immobilization or resection of diseased tissues.^26^ Such methods, for example, arthroplasty and spinal fusion, are costly and invasive surgeries that come with a high risk of adjacent segment disease, involving the promotion of degeneration in neighboring IVDs.^27^ Moreover, the long‐term benefits in pain alleviation and disability reduction remain controversial.^28^, ^29^, ^30^ Despite stated limitations, these surgeries are becoming more frequent and are speculated to continue to increase drastically in the coming years.^31^ Therefore, new methods of intervention are highly desired, specifically, methods to alleviate discogenic pain to prevent or extend the need for surgery as well as agents that target the underlying pathophysiology of LBP. Thus, regenerative strategies e.g., tissue engineering,^32^, ^33^ growth factor injection,^34^, ^35^ extracellular vesicles,^36^, ^37^, ^38^ biomaterials,^39^, ^40^ and gene therapy^41^ are highly anticipated techniques. Specifically, cell therapy^42^ and platelet‐rich plasma (PRP)^43^ derived products are posed as a golden opportunity owing to its proposed ability to tackle the decline in cell function and cell numbers that are fundamental to the degenerative cascade and discogenic pain (Figure 1c,d). Multiple in vivo studies have emphasized the strong capacity of intradiscal cell injection to limit, halt, or reverse induced disc degeneration features.^44^ Since then, multiple in human studies have attempted to alleviate discogenic pain and disability and promote regeneration of disc features in naturally occurring disc degeneration. These clinical trials and case reports have been summarized by multiple reviews concluding a trend of beneficial effects.^42^, ^45^, ^46^, ^47^, ^48^, ^49^ Nonetheless, these reviews are highly speculative and fail to fully present the scale and impact of the claimed “improvements.” For a comprehensive understanding of their impact, it is crucial to evaluate not only statistically significant improvements but also their clinical significance. Furthermore, improvements are ideally compared to control conditions (e.g., placebo controls or standard of care, e.g., spinal fusion surgery) to fully identify their added value.^50^ Only a single meta‐analysis by Wu et al.^51^ on this topic has been published, which underscores the potential of cellular therapeutics. Nevertheless, this review was severely limited by the small number (*N* = 6) of eligible controlled and randomized clinical trials. Moreover, the reported outcomes were not discussed in a frame of clinical significance. As such, a need exists to comprehensively examine the overall improvements reported by cell or PRP therapy against discogenic pain and determine whether these observations are productive in a clinical setting and competitive with standard methods of care.

With this systematic review, we aim to capture the full course of pain and disability changes for discogenic LBP patients treated with investigational cellular and PRP therapeutics, presenting the complete observation of changes from baseline scores to final follow‐up and assessing their clinical significance. Moreover, by comparing the different trends in pain relief against patient cohorts treated with spinal fusion, we aim to identify the impact of regenerative cellular treatment strategies and their value as prospective orthopedic medical agents.

## 2 METHODS

### 2.1 Systematic literature search and screening

**FIGURE 2:**
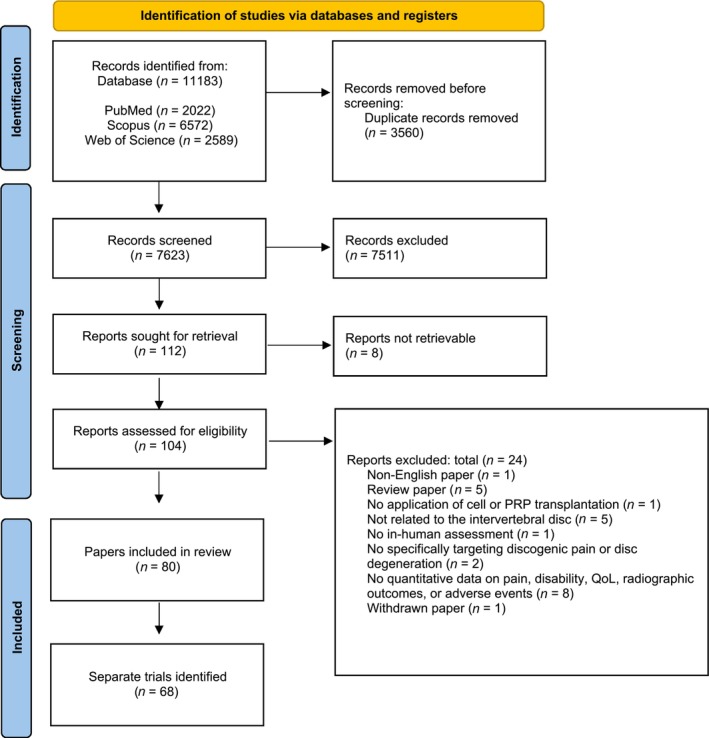
Modified PRISMA 2020 flow diagram. Overview of our systematic review presenting the retrieval, screening, and selection of papers and the subsequent identification of separate clinical trials.

Following prospectively registered protocols (PROSPERO: CRD42023437508)^52^ and PRISMA guidelines,^53^ the initial phase of this review involved a comprehensive systematic search of PubMed, Scopus, and Web of Science online databases (date of search: July 7, 2023) employing the syntax presented in Data S1, Supporting Information. Two researchers (ST and JS) independently screened all articles using RAYYAN software.^54^ Only reports in English that (1) involved transplantation of a cellular or PRP (derivative) product as the primary therapeutic, (2) applied to affect (in part) the IVD, (3) involved in‐human assessment, and (4) described safety and/or efficacy assessments were included. Safety assessment involved reporting the number or rate of serious adverse events (SAEs) following transplantation. Efficacy reporting required the description or graphical presentation of pain (visual analog score [VAS], numerical rating scale [NRS], numerical pain scale [NPS]), disability (Oswestry Disability Index [ODI]), quality of life (QoL) outcomes (Short form [SF]‐12 or SF‐36), and/or radiographic outcomes (T2 intensity, apparent diffusion coefficient [ADC], Pfirrmann grade, disc height) with (calculable) average values of absolute outcomes or change from baseline outcomes. Screening was performed based on article title and abstract, followed by final full text assessment (Figure 2). Papers were excluded if the product was employed to induce spinal fusion or treat cancer.

### 2.2 Data collection and presentation

The literature was analyzed to determine which reports described identical studies, for example, the same trial but different follow‐up times (see Table 1; for full reference list see Data S2). General study aspects were recorded, such as study type and location, patient inclusion, and exclusion criteria (including discography, radiculopathy, magnetic resonance imaging (MRI) confirmed degeneration, herniation, high intensity zones, and Modic changes), products transplanted (including the cell types, volumes, carriers, and cell numbers), and transplantation methods (such as injection location and needle size). Next, all VAS, NPS, or NRS pain scores, ODI disability scores, SF12, SF36, MRI Pfirrmann grade, ADC, disc height, MRI intensity outcomes, and SAE were collected from the included studies and were recorded independently by two reviewers (ST and JS). Data were collected separately for different treatment groups within one trial (e.g., high‐ and low‐dose cohorts or different injection site cohorts). Specifically, data were carefully collected from the main text, tables, or supplementary items. Additionally, data presented in graphs were analyzed using WebPlotDigitizer version 4.4 (https://automeris.io/WebPlotDigitizer, by A. Rohatgi) to retrieve an estimate of reported scores. VAS scores presented in mm were divided by 10 to match the range of scores given in cm. Finally, the average pain scores, ODI scores, and changes in pain and ODI scores were determined for baseline and 1‐, 3‐, 6‐, 12‐, and 24‐month post‐transplantation follow‐ups. Here, the average value was determined by calculating the average of each trial's reported pain or ODI score at each time point, multiplied by the number of analyzed patients. If the number of patients was not specifically mentioned for each time point, the general recruited patient number was included. Changes in pain and ODI scores were then compared to rates for spinal fusion surgery as reported by Koenders et al.^55^ Additionally, changes were determined to meet minimal clinically important difference (MCID) standards, in which a change in pain of 2.5 points (based on Ostelo & de Vet^56^), 12.8 (based on Johnsen et al.^57^), or 17 ODI points (based on Maughan & Lewis^58^) was considered clinically significant. The results were analyzed for both cell therapy and PRP‐product injections separately as well as collectively. Finally, the risk of bias was assessed independently by both reviewers using the methodological index for nonrandomized studies (MINORS) scheme.^59^ Additionally, for randomized controlled trials, the Cochrane Source of Bias tool was employed.^60^ Final presentation of collected data was realized using GraphPad Prism v10.0.2 (GraphPad Software LLC) and Adobe Illustrator version 27.8.1 (Adobe Inc.).

**TABLE 1:** Tabular overview of identified clinical trials and case reports on cell‐ and PRP‐based therapeutics (see Data S2 for reference overview).

Cell type	Author	Reporting type	FU (max)	Registration ID	Product (s)	*n* (max)	Control (s)	*n* (max)	Efficacy outcomes	Safety outcomes
Chondrogenic cells	Coric	Phase I	1	BB‐IND13985	AC	15	‐	‐	✔	✔
	Ruan	Pilot study	6	‐	IVD allograft	5	‐	‐	✔	✔
	Zhang	Retrospective	>6	‐	IVD allograft	25	Fusion surgery	43	✔	✔
	Meisel	Pilot study	2	‐	IVD‐C	12	Discectomy	16	✔	✔
	Tschugg	Phase I	<0.5	EudraCT2010‐023830‐22, NCT01640457	IVD‐C	12	Discectomy + HA‐PEG (Carrier)	12	✘	✔
	Schwan	Retrospective	NA	‐	IVD‐C	10	Discectomy	10	✘	✔
	Xuan	Pilot study	6	‐	IVD‐C	18	Discectomy	22	✔	✔
	Mochida	Phase I	3	‐	NPC	9	‐	‐	✔	✔
	Hunter	Pilot study	1	NCT03709901	NPC	140 (+35)^a^	Saline (Placebo)	39	✔	✔
							Conservative care	39		
Mesenchymal stromal cells	Jung	Case report	<0.5	‐	AD‐MSC	1	‐	‐	✘	✔
	Piccirilli	Case series	1	‐	AD‐MSC	2	‐	‐	✘	✔
	Kumar	Phase I	1	NCT02338271	AD‐MSC	10	‐	‐	✔	✔
	Bates	Pilot study	1	‐	AD‐MSC	9	‐	‐	✔	✔
	Orozco	Phase I	1	EudraCT2008‐001191‐68	BM‐MSC	10	‐	‐	✔	✔
	Noriega	Phase I/II	3.5	EudraCT2012‐004444‐30, NCT01860417	BM‐MSC	12^b^	Paravertebral muscle anesthetic	12^b^	✔	✔
	Papadimitriou	Pilot study	2	‐	BM‐MSC	10	‐	‐	✔	✔
	Amirdelfan	Phase I/II	3	NCT01290367	MPC	60	HA vehicle (Carrier) Saline (Placebo)	20 20	✔	✔
	Lewandrowski	Retrospective	2	‐	UC‐MSC	33	‐	‐	✔	✔
	Pang	Case series	2	‐	UC‐MSC	2	‐	‐	✔	✔
Cocktail of cell types	Xu	Pilot study	2	NCT03002207	BMA	15	Discectomy	15	✔	✔
							Discectomy + AF suture	15		
	Atluri	Pilot study	1	‐	BMC	40	Conventional treatment	40	✔	✔
	Haines	Pilot study	1	‐	BMC	32	‐	‐	✔	✔
	Pettine	Pilot study	3	‐	BMC	26	‐	‐	✔	✔
	Wolff	Retrospective	1	‐	BMC	33	‐	‐	✔	✔
	El‐Kadiry	Case series	1	‐	BMC	18	‐	‐	✔	✔
	Jerome	Case series	<0.5	‐	BMC	3	‐	‐	✘	✔
Platelet‐rich plasma products	Centeno	Retrospective	2	‐	PL	470	‐	‐	✔	✔
	Akeda	Pilot study	1	UMIN000038536jRCTs043190014	PL	9 (+6)^a^	Corticosteroids	7 (−6)^a^	✔	✔
	Akeda	Pilot study	5.9	‐	PL	11	‐	‐	✔	✔
	Kirchner	Case report	3		LP‐PRP	1	‐	‐	✔	✘
	Kirchner	Retrospective	0.5	‐	LP‐PRP	86	‐	‐	✔	✔
	Kirchner	Case report	0.5	‐	LP‐PRP	1	‐	‐	✔	✔
	Beatty	Case report	1	‐	LP‐PRP	1	‐	‐	✘	✔
	Bise	Pilot study	<0.5	‐	LP‐PRP	30	Epidural steroid	30	✔	✔
	Kirchner	Retrospective	2	‐	LP‐PRP	65	‐	‐	✘	✔
	Zielinski	Pilot study	<0.5	‐	LP‐PRP	18	Saline (Placebo)	8	✘	✔
	Li	Pilot study	0.5	‐	LP‐PRP	25	Discectomy	25	✔	✘
							Discectomy + AF suture	25		
	Zhang	Pilot study	1	ChiCTR1900024268	LP‐PRP	31	‐	‐	✔	✔
	Lam	Case report	0.8	‐	LP‐PRP	1	‐	‐	✔	✔
	Le	Pilot study	1	‐	LP‐PRP or LR‐PRP	25	‐	‐	✘	✔
	Levi	Pilot study	0.5	‐	LR‐PRP	22	‐	‐	✔	✔
	Cheng	Unspecified	>6	‐	LR‐PRP	19	‐	‐	✔	✘
	Ruiz‐Lopez	Pilot study	0.5	‐	LR‐PRP	25	Corticosteroid	25	✔	✔
	Jain	Pilot study	0.5	CTRI/2019/05/019434	LR‐PRP	20	‐	‐	✔	✔
	Lam	Case series	<0.5	‐	LR‐PRP	3	‐	‐	✔	✔
	Kawabata	Case report	0.5	jRCTb042210159	LR‐PRP	2	‐	‐	✔	✔
	Monfett	Pilot study	2	‐	PRP _(unspecified)_	29 (+18)^a^	Contrast agent	18 (−18)^a^	✔	✔
	Bhatia	Pilot study	<0.5	‐	PRP _(unspecified)_	10	‐	‐	✔	✔
	Demirci	Retrospective	1.2	‐	PRP _(unspecified)_	31	Corticosteroids	31	✔	✔
	Navani	Case series	0.5	‐	PRP _(unspecified)_	6	‐	‐	✔	✔
	Karamanakos	Case report	3	‐	PRP _(unspecified)_	1	‐	‐	✔	✘
	Singh	Pilot study	0.5	CTRI/2020/09/027879	PRP _(unspecified)_	23	Steroid	22	✔	✘
	Wongjarupong	Pilot study	0.5	NCT05234840	PRP _(unspecified)_	15	Steroid	15	✔	✔
	Saraf	Pilot study	0.5	‐	PRP _(unspecified)_	29	Steroid	31	✔	✔
	Lutz	Case report	1	‐	PRP _(unspecified)_	1	‐	‐	✘	✔
	Lam	Case report	<0.5	‐	PRP _(unspecified)_	1	‐	‐	✔	✔
	Wu	Case report	<0.5	‐	PRP _(unspecified)_	2	‐	‐	✔	✔
	Xu	Pilot study	1	CTR17011825	PRP _(unspecified)_	61	Steroid injection	63	✘	✔
	Jiang	Pilot study	1	CTR1800017228	PRP _(unspecified)_	51	Discectomy	57	✔	✔
	Godek	Retrospective	0.5	‐	PRP _(unspecified)_	108	‐	‐	✘	✔
	Lutz	Retrospective	3.6	‐	PRP _(unspecified)_	66	‐	29	✔	✔
	Rawson	Case report	<0.5	‐	PRP _(unspecified)_ + PL	2	‐	‐	✘	✔
	Williams	Case series	2	‐	PL + PRP _(unspecified)_ and/or PPP	9	‐	‐	✔	✔
Other	Subach	Case report	1	‐	AT, BMA, plasma	1	‐	‐	✘	✔
	Centeno	Pilot study	6	NCT03011398	BM‐MSC + PL	25^c^	‐	‐	✔	✔
	Ramos	Case report	1	‐	BMC + PRP _(unspecified)_	1	‐	‐	✘	✔
	Comella	Pilot study	1	NCT02097862	SVF + PRP _(unspecified)_	15	‐	‐	✔	✔
	Singh	Case report	<0.5	‐	“Stem cells”	1	‐	‐	✘	✔

Abbreviations: AF, annulus fibrosis; AC, articular chondrocyte; AD‐MSC, adipose‐derived mesenchymal stromal cells; AT, adipose tissue; BMA, bone marrow aspirate; BMC, bone marrow concentrate; BM‐MSC, bone marrow mesenchymal stromal cell; FU, follow‐up (in years); HA, hyaluronic acid; IVD‐C, intervertebral disc cells; LP‐PRP, leucocyte poor platelet‐rich plasma; LR‐PRP, leucocyte‐rich platelet‐rich plasma; MPC, mesenchymal precursor cells; NA, not applicable; NPC, nucleus pulposus cell; PEG, polyethylene glycol; PL, platelet lysate; PPP, platelet poor plasma; PRP, platelet‐rich plasma; SVF, stromal vascular fraction; UC‐MSC, umbilical cord mesenchymal stromal cells.

^a^ Crossover.

^b^ Patient numbers at final follow‐up unclear.

^c^ Patient number analyzed in the paper.

## 3 RESULTS

### 3.1 Systematic literature search and screening

**FIGURE 3:**
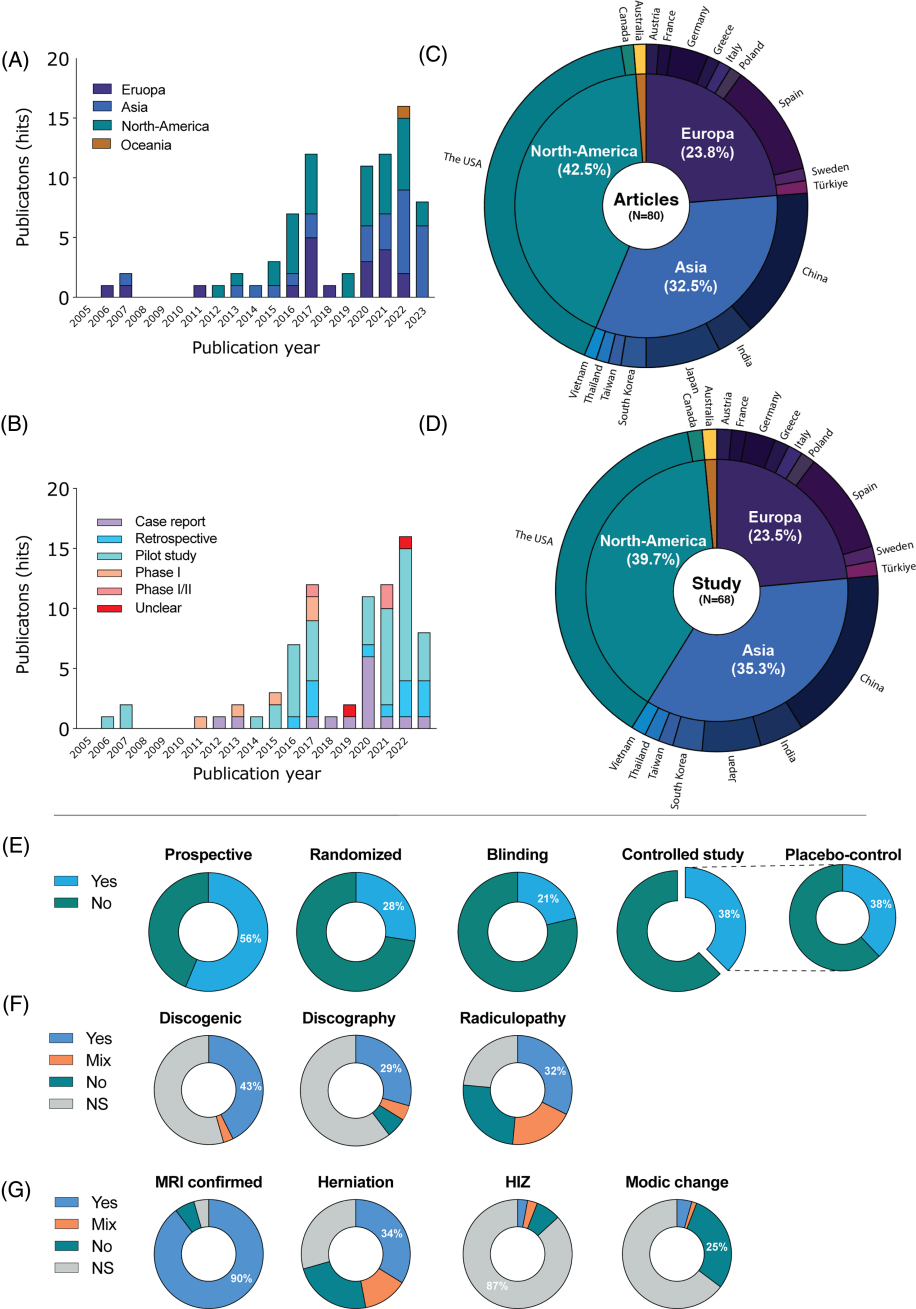
Figure panel presenting trends in cell‐ and PRP therapy trials and publications. All identified papers sorted per year of publication and (A) continents of origin and (B) type of report. Pie charts presenting the rate of (C) publications and (D) separate trials reported per country and continent. Pie charts representing the proportions of (E) prospective, randomized, blinded (in any form), and controlled and subsequential placebo‐controlled trials identified in our systematic review. Pie charts presenting indications regarding patients including with regard to (F) pain (states discogenic pain, applied discography to confirm painful disc, or presence of radiculopathy) and (G) disc degeneration (MRI confirmed, presence of disc herniation, high intensity zones, and Modic changes). HIZ, high intensity zone; MRI, magnetic resonance imaging.

Our systematic review yielded a total of 11 183 papers, of which 7623 papers were manually determined as non‐duplicates. Subsequent title and full‐text screening found 80 papers to match our inclusion criteria (Figure 2; see Data S2 for reference list). Further assessment determined that these 80 papers comprised a total of 68 separate clinical trials/studies. This encompassed a (minimum) total of 1974 cell/PRP‐treated patients. The frequency of publications showed an overall increase over time (Figure 3a,b). The largest number of publications originated from North America (42.5%), where Asian and European countries presented 32.5% and 23.8% of reports, respectively (Figure 3c). This trend is slightly nuanced when examining the number of separate trials (Table 1 and Figure 3d). The identified trials were marked as prospective in 56% of reports (Figure 3e). Randomization and blinding were only reported in 28% and 21% of articles, respectively. Moreover, only approximately one‐third of trials included a control group, of which only 38% employed a placebo cohort. Of the 68 studies identified, 52 (76.5%) included some efficacy outcomes, and 63 studies (92.6%) recorded outcomes on adverse events (Table 1). The overall risk of bias assessment revealed a general high risk of bias for the included studies (Data S3).

The included studies recruited a wide range of patients; however, notably, specific aspects of disc degeneration and the diagnosis of LBP were often not specified. Notably, with regard to pain indications, only 43% of trials specified LBP as discogenic, with 54% of trials making no such specification (Figure 3f). Here, only 29% of studies employed discography to confirm the pain source of their cohorts, while 6% had listed discography as an exclusion criterion. Approximately a quarter of studies included radiculopathy as an inclusion criterion, while equal parts stated it as an exclusion criterion, had a mixed selection, or did not specify this aspect of their patient cohort. Regarding disc degeneration, a large majority (90%) of studies required disc degeneration to be confirmed via MRI (Figure 3g). Disc herniation was used as an inclusion criterion for 34% and as an exclusion criterion for 24% of studies. High‐intensity zones or Modic changes were most often not specified but were used as exclusion criteria in a minority of studies.

**FIGURE 4:**
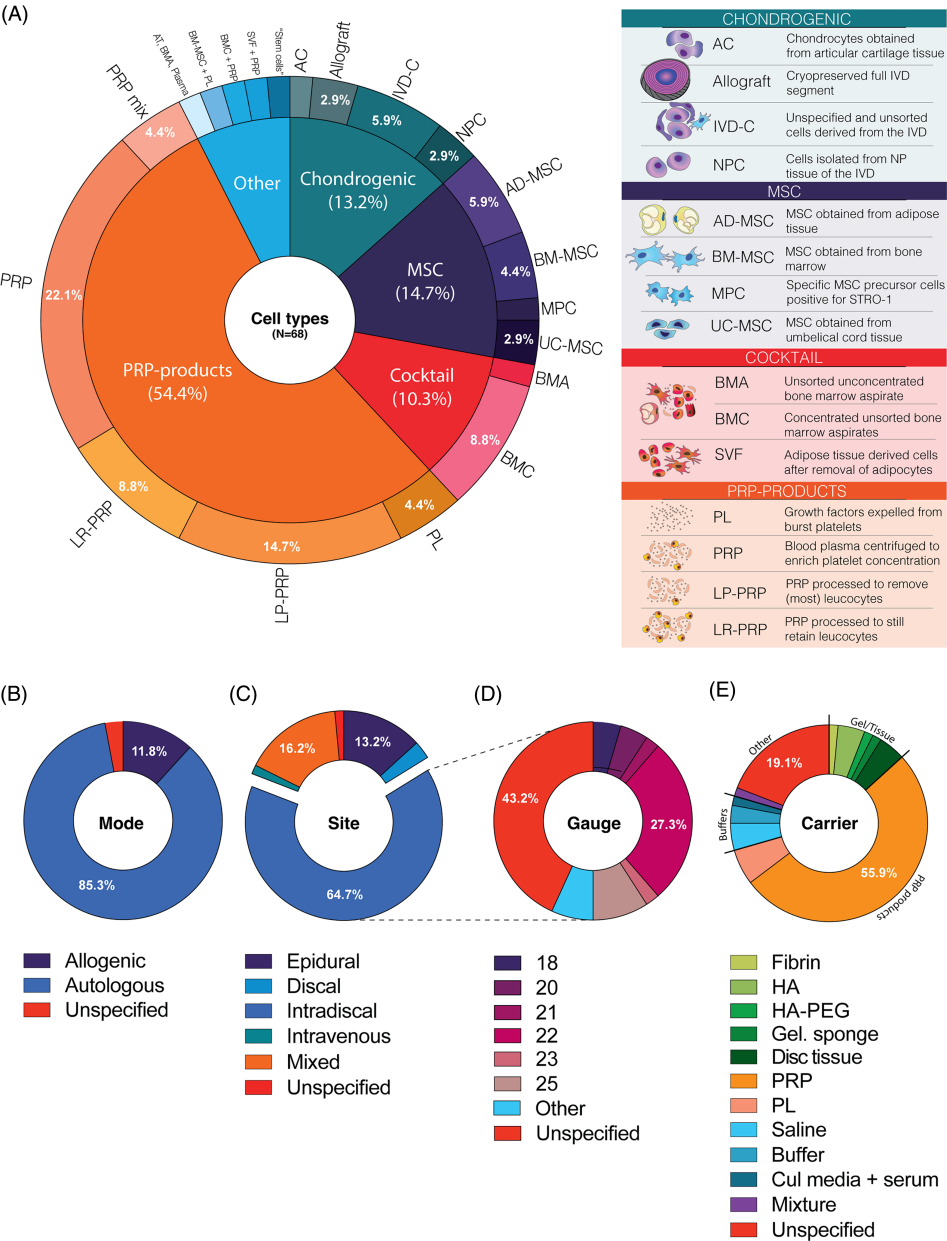
Figure panel presenting trends in cell‐ and PRP products and transplantation strategies. (A) Pie chart of the rate of different cell types employed in the identified studies. Pie charts of (B) mode, (C) injection site, (D) gauge size of intradiscal products, and (E) carrier employed for transplantation. AC, articular cartilage cells; AD‐MSC, adipose‐derived mesenchymal stromal cells; BMA, bone marrow aspirate; BMC, bone marrow concentrate; BM‐MSC, bone marrow mesenchymal stromal cell; Cul media, cell culture media; Gel sponge, gelatin sponge; HA, hyaluronic acid; IVD‐C, intervertebral disc cells; LP‐PRP, leukocyte poor/free platelet‐rich plasma; LR‐PRP, leucocyte‐rich platelet‐rich plasma; MPC, mesenchymal precursor cells; NP, nucleus pulposus; NPC, nucleus pulposus cell; PEG, Poly(ethylene glycol); PL, platelet lysate; PRP, platelet‐rich plasma; SVF, stromal vascular fraction; UC‐MSC, umbilical cord mesenchymal stromal cells.

The included studies employed a wide variety of different products and transplantation strategies (Data S4). The most common products were general PRP products, representing 54.4% of the identified studies (Figure 4a). Next was the transplantation of mesenchymal stromal cell (MSC)‐based products (14.7%), followed by chondrogenic cells (13.2%). In addition, 10.3% of studies employed a direct bone marrow aspirate (BMA) product. Considering the high rate of studies applying PRP products, unsurprisingly, the rate of autologous transplantation products was high at 85.3% (Figure 4b). The largest number of studies applied their product through intradiscal injection (64.7%), whereas some PRP products were also administered epidurally (13.2%) (Figure 4c). For the intradiscal injection, needle gauge size was often not specified (43.2%; especially for PRP products), and the most commonly reported needle size was 22 G in 27.3% of studies (Figure 4d). A specialized carrier was not commonly employed, that is, injection of a PRP‐derived product (55.9%) or a saline solution (4.4%) (Figure 4e). Alternatively, hyaluronic acid (HA)‐based gels at 5.9% or disc tissue grafts at 4.4% represented the most common carriers. Notably, 19.1% of reports did not specify the transplantation carrier.

### 3.2 Pain alleviations

**FIGURE 5:**
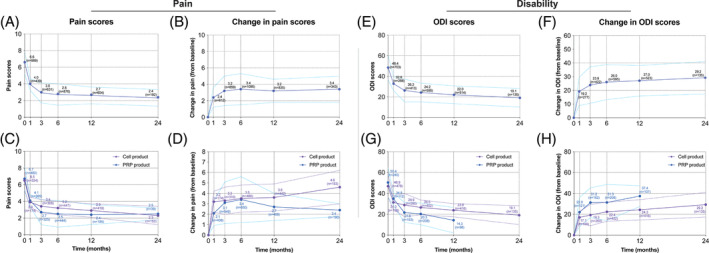
Trends in pain and ODI alleviation following cell and platelet‐rich plasma (PRP) transplantation. (A) General trends of pain scores in identified trial weighted by sample size and similarly (b) the trend in pain score changes reported. (C, D) General trends in pain and pain alleviation for PRP and cell‐therapies. (E) General trends of ODI scores in identified trial weighted by sample size and similarly (F) the trend in ODI score changes reported. (G, H) General trends in ODI and ODI change for PRP and cell‐therapies. Dots represent average values recorded at indicated time point and dashed line indicates 95% confidence interval.

Of the identified studies, 49 separate trials reported pain outcomes based on VAS, NRS, or NPS (Table 2 and Data S5). All studies were able to report a trend of enhanced pain reduction, with most studies able to maintain their obtained alleviation for the duration of the follow‐up (Data S5a). The exceptions are the studies by Ruan et al.,^61^ Ruiz‐Lopez et al.,^62^ and the spinal chain injection group for El‐Kadiry et al.^63^ No clear differences between the cell and PRP‐treated cohorts were observed (Data S5c,e). The median average baseline score was 6.5 and ranged from 0.9 to 9.0 (Table 2). These showed a reduction of a median of 3.7 points, ranging from −0.5 to 9.0 at the final follow‐up. The clear exception was the work of Meisel et al.,^64^, ^65^ in which cells were injected after discectomy, which was already able to promote most pain alleviation prior to cell injection, resulting in a baseline score of 1.9. Similarly, Ruan et al.^61^ started with an average 0.9 neck pain score in their 5‐patient cohort; the relatively low baseline levels resulted in overall worsening of pain levels at their 72‐month follow‐up. Excluding these two studies, the overall pain reduction of the other trials ranged from 1.2 to 9.0, with a median of 3.8 points (Table 2). This equates to an estimated median relative change of 63.8%, with a range of 15.0%–100%, with no clear differences between PRP or cell‐treated cohorts (Data S5). More specifically, a median change of 3.4 (range: 1.1–9.0) and 3.8 (range: 0.6–7.0) points could be determined at the 6‐ and 12‐month follow‐ups, respectively. Estimating the overall average pain and pain reduction values by weighing each study result by the cohort sample size, the results in the data presented in Figure 5a–d suggest that with an average baseline pain score of 6.6 points, a 2.8 score at 6 months, a 2.7 score at 12 months, and a 2.4 score at 24 months can be expected (Figure 5a; based on 876, 604, and 192 patients, respectively). Alternatively, a pain reduction of 3.4 (*N* = 1095) at 6 months, 3.2 (*N* = 835) at 12 months, and 3.4 (*N* = 343) at 24 months was observed (Figure 5b). Our data suggest an overall higher trend for cell‐based treatments (Figure 5c), where cell‐treated cohorts resulted in final pain reductions of 3.5, 3.6, and 4.6 at 6 months, 12 months, and 24 months, respectively (Figure 5d). Compared to 3.4, 2.7, and 2.4 for PRP‐treated cohorts. In contrast, MCID comparisons suggested that MSC and chondrogenic cell therapies (except for Meisel et al.^64^, ^65^ and Ruan et al.^61^) were all able to report clinically significant improvements, while BMC and PRP products showed varying results (Table 2). Overall, cell‐based products (excluding Meisel et al. and Ruan et al.) achieved MCID in 16 out of 21 (76%) and 16 of 21 (76%) differently treated cohorts at the 6‐ and 12‐month follow‐ups, respectively. This was a markedly higher rate than PRP‐based products, with 12 out of 20 (60%) and 7 out of 12 (58%) study cohorts achieving MCID at the 6‐ and 12‐month follow‐ups, respectively. If MCID success rates were compared for each study at their final follow‐up, a rate of 78% (40 out of 51) can be reported.

**TABLE 2:** Tabular overview of reported pain improvements.

Cell type	Specifications			6 months			12 months			Final follow‐up				
	Author	Score type	Baseline pain	Pain	Pain change	MCID^a^	Pain	Pain change	MCID^a^	FU (months)	Pain	Pain change	Change (%)^b^	MCID^a^
Chondrogenic cells	Coric	NRS	5.7	0.8	4.9	✔	3.1	2.6	✔	12	3.1	2.6	46%	✔
	Ruan	VAS	0.9	‐	‐	‐	‐	‐	‐	72	1.4	−0.5	−56%	✘
	Zhang	VAS	6.4	0.6	5.8	✔	2.6	3.8	✔	84	1.2	5.2	81%	✔
	Meisel^c^	VAS	1.9^c^	2.1	−0.2	✘^c^	1.8	0.1	✘^c^	24	1.1	0.8^c^	42%	✘^c^
	Xuan^d^	VAS	4.9	1.7	3.2	✔	1.4	3.5	✔	72	1.2	3.7	76%	✔^d^
	Hunter	VAS	6.5	3.3	3.2	✔	3.0	3.5	✔	12	3.0	3.5	54%	✔
Mesenchymal stromal cells	Kumar (Low)	VAS	6.4	3.6	2.8	✔	2.2	4.2	✔	12	2.2	4.2	66%	✔
	Kumar (High)	VAS	6.4	3.0	3.4	✔	3.8	2.6	✔	12	3.8	2.6	41%	✔
	Bates	VAS	‐	‐	4.2	✔	‐	4.3	✔	12	‐	4.3	‐	✔
	Orozco	VAS	6.9	2.2	4.7	✔	2.0	4.9	✔	12	2.0	4.9	71%	✔
	Noriega	VAS	6.7	4.0	2.7	✔	4.7	2.0	✘	42	3.0	3.7	56%	✔
	Papadimitriou	NRS	7.4	6.1	1.3	✘	4.3	3.1	✔	24	4.6	2.8	38%	✔
	Amirdelfan (Low)	VAS	7.0	2.6	4.4	✔	3.1	3.9	✔	36	3.6	3.4	49%	✔
	Amirdelfan (High)	VAS	7.1	3.5	3.6	✔	3.2	3.9	✔	36	2.8	4.3	61%	✔
	Lewandrowski	VAS	8.2	2.0	6.2	✔	1.8	6.4	✔	24	1.7	6.6	80%	✔
	Pang	VAS	7.5	2.0	5.5	✔	2.0	5.5	✔	24	2.5	5.0	67%	✔
Cocktail of cell types	Xu^d^	VAS	7.4	2.0	5.4	✔^d^	1.5	5.9	✔^d^	24	1.4	6.1	82%	✔^d^
	Atluri	NRS	7.1	3.7	3.4	✔	4.2	2.9	✔	12	4.2	2.9	41%	✔
	Haines	VAS	5.4	‐	‐	‐	3.0	2.4	✘	12	3.0	2.4	44%	✘
	Pettine	VAS	8.2	‐	‐	‐	3.3	4.9	✔	36	2.2	5.7	73%	✔
	Wolff	NRS	5.2	3.9	1.3	✘	3.9	1.3	✘	12	3.9	1.3	25%	✘
	El‐Kadiry (Intradiscal)	VAS	6.0	3.4	2.6	✔	2.2	3.8	✔	12	2.2	3.8	63%	✔
	El‐Kadiry (PSCI)	VAS	8.0	6.4	1.6	✘	6.8	1.2	✘	12	6.8	1.2	15%	✘
Platelet‐rich plasma products	Centeno	NPS	5.1	3.2	1.9	✘	3.0	2.1	✘	24	2.5	2.6	51%	✔
	Akeda (Non‐crossover)	VAS	6.8	‐	‐	‐	1.2	5.6	✔	12	1.2	5.6	82%	✔
	Akeda (Crossover)	VAS	5.8	‐	‐	‐	2.1	3.7	✔	12	2.1	3.7	64%	✔
	Akeda	VAS	7.5	‐	‐	‐	2.9	4.6	✔	72	2.1	5.4	72%	✔
	Kirchner	VAS	8.4	0.8	7.6	✔	‐	‐	‐	6	0.8	7.6	91%	✔
	Kirchner	NRS	9.0	0.0	9.0	✔	‐	‐	‐	6	0.0	9.0	100%	✔
	Bise	NRS	6.3	‐	‐	‐	‐	‐	‐	1.5	4.0	2.3	37%	✘
	Li	NRS	8.2	1.0	7.2	✔	‐	‐	‐	6	1.0	7.2	88%	✔
	Zhang	NRS	5.6	3.7	1.9	✘	3.4	2.2	✘	12	3.4	2.2	39%	✘
	Lam	NRS	7.5	‐	‐	‐	‐	‐	‐	10	0.5	7.0	93%	✔
	Levi	VAS	6.6	4.1	2.5	✘	‐	‐	‐	6	4.1	2.5	37%	✘
	Cheng	NRS	4.7	‐	‐	‐	3.1	1.6	✘	78	1.3	3.4	72%	✔
	Ruiz‐Lopez	VAS	7.5	6.1	1.4	✘	‐	‐	‐	6	6.1	1.4	19%	✘
	Jain	NRS	5.9	3.1	2.8	✔	‐	‐	‐	6	3.1	2.8	47%	✔
	Lam	VAS	6.5	‐	‐	‐	‐	‐	‐	0.8	0.5	6.0	92%	✔
	Kawabata	VAS	6.0	4.5	1.5	✘	‐	‐	‐	6	4.5	1.5	25%	✘
	Monfett	NRS	4.7	3.6	1.1	✘	3.2	1.6	✘	24	2.4	2.3	50%	✘
	Bhatia	VAS	6.1	‐	‐	‐	‐	‐	‐	3	3.7	2.4	39%	✘
	Demirci	VAS	8.9	4.2	4.7	✔	3.8	5.1	✔	15	3.8	5.1	57%	✔
	Navani	VPS	4.8	0.2	4.6	✔	‐	‐	‐	6	0.2	4.6	96%	✔
	Karamanakos	VAS	8.0	‐	‐	‐	1.0	7.0	✔	12	1.0	7.0	88%	✔
	Singh	NRS	6.9	1.4	5.5	✔	‐	‐	‐	6	1.4	5.5	80%	✔
	Wongjarupong	VAS	6.4	1.8	4.6	✔	‐	‐	‐	6	1.8	4.6	72%	✔
	Saraf	VAS	6.7	3.5	3.2	✔	‐	‐	‐	6	3.5	3.2	48%	✔
	Wu	VAS	7.5	‐	‐	‐	‐	‐	‐	2	1.5	6.0	80%	✔
	Jiang	VAS	5.0	0.9	4.1	✔	0.8	4.2	✔	12	0.8	4.2	84%	✔
	Lam	VAS	7.5	‐	‐	‐	‐	‐	‐	1	1.5	6.0	80%	✔
	Williams	NPS	4.9	2.0	2.9	✔	1.7	3.2	✔	24	1.6	3.3	67%	✔
Other	Centeno	NPS	‐	‐	1.4	✘	‐	0.6	✘	72	‐	3.3	‐	✔
	Comella	VAS	5.6	3.6	2.0	✘	‐	‐	‐	6	3.6	2.0	36%	✘

*Note*: Table of all identified studies and a summarizing statement regarding the reported pain scores and pain improvements at the final follow‐up time point.

Abbreviations: AC, articular chondrocyte; AD‐MSC, adipose‐derived mesenchymal stromal cells; BMA, bone marrow aspirate; BMC, bone marrow concentrate; BM‐MSC, bone marrow mesenchymal stromal cells; FU, follow‐up (in years); IVD‐C, intervertebral disc cells; LP‐PRP, leucocyte poor platelet‐rich plasma; LR‐PRP, leucocyte‐rich platelet‐rich plasma; MCID, minimally clinical important difference; MPC, mesenchymal precursor cells; NPC, nucleus pulposus cell; NRS, numerical rating scale; PL, platelet lysate; PRP, platelet‐rich plasma; PSCI, posterior spinal chain injection; SVF, stromal vascular fraction; UC‐MSC, umbilical cord mesenchymal stromal cells; VAS, visual analogue scale.

^a^ Following recommendations of Ostelo and de Vet.^56^

^b^ Estimated change as percentage from baseline with cell color representing low (red = 0%) to high (blue = 100%) reduction in pain outcomes.

^c^ Involves cell transplantation after discectomy has already been able to provide pain relief.

^d^ Cell transplantation was performed concurrently with microdiscectomy.

### 3.3 Disability recovery

For our review, 32 separate trials were identified (Table 3) that reported on ODI improvements. All studies were able to report some degree of ODI reduction with different degrees of success (Data S6a,b). From a median baseline ODI (excluding Meisel et al.^64^, ^65^) of 46.1 (range: 25.0–76.6), a median ODI of 23.3 (range: 2.0–45.4), and 22.0 (range: 4.3–34.4) was achieved at the 6‐ and 12‐month follow‐ups, respectively (Table 3 and Data S6a). This equates to a median change of 24.0 (range: 1.0–62.3) and 27.2 (range: 10.0–52.0) ODI at the 6‐ and 12‐month follow‐ups, respectively. Again, the trends between PRP and cell‐treated cohorts were similar, although PRP trials did not include any results after 12 months of follow‐up (Data S6). Estimating the overall average ODI and ODI reduction values by weighing each study result by the cohort sample size (Figure 5e–h) showed an overall trend of ODI reduction from baseline score of 48.4 24.2 (*N* = 595), 22.0 (*N* = 514), and 19.1 (*N* = 135) at 6, 12, and 24 months, respectively (Figure 5e). Alternatively, 26.0 (*N* = 595), 27.0 (*N* = 523), and 29.2 (*N* = 135) ODI reductions at 6, 12, and 24 months, respectively, were calculated (Figure 5f). Here, PRP treatment appeared to result in slightly enhanced improvements compared to cell‐treated cohorts, although it should be considered that the results were derived from a small selection of studies with small sample sizes, as well as missing long‐term follow‐up results (Figure 5g,h). Specifically, PRP products showed an overall reduction of 31.3 (*N* = 208) and 37.4 (*N* = 107) ODI at 6 and 12 months, respectively, compared to 22.4 (*N* = 402) and 24.3 (*N* = 416) for cell‐based products. In contrast, MCID comparisons (excluding Meisel et al.) resulted in similar rates of success between cell‐ and PRP‐based products. Cell therapeutics reported MCID for 12 out of 18 (67%) and 13 out of 18 (72%) treated studies at 6 and 12 months, respectively, while PRP reported 7 out of 10 (70%) and 4 out of 5 (80%) (Table 3). If MCID success rates are compared for each study at their final follow‐up, a rate of 76% (26 out of 34) was reported.

**TABLE 3:** Tabular overview of reported disability improvements.

Cell type	Specifications		6 months			12 months			Final follow‐up				
	Author	Baseline ODI	ODI	ODI change	MCID^a^	ODI	ODI change	MCID^a^	FU (months)	ODI	ODI change	Change (%)^b^	MCID^a^
Chondrogenic cells	Coric	53.3	26.9	26.4	✔	20.3	33.0	✔	12	20.3	33.0	62%	✔
	Meisel^c^	16.1	18.6	−2.5	✘^c^	15.6	0.5	✘^c^	24	12.0	4.1	26%	✘^c^
	Xuan^d^	73.0	45.4	27.6	✔^d^	34.4	38.6	✔^d^	72	24.1	48.9	67%	✔^d^
	Hunter	51.2	28.1	23.1	✔	24.1	27.1	✔	12	24.1	27.1	53%	✔
Mesenchymal stromal cells	Kumar (Low)	37.2	19.4	17.8	✔	15.2	22.0	✔	12	15.2	22.0	59%	✔
	Kumar (High)	50.0	23.2	26.8	✔	22.8	27.2	✔	12	22.8	27.2	54%	✔
	Bates	‐	‐	34.0	✔	‐	39.0	✔	12	‐	39.0	‐	✔
	Orozco	25.0	9.4	15.6	✘	7.4	17.6	✔	12	7.4	17.6	70%	✔
	Noriega	34.0	20.0	14.0	✘	22.0	12.0	✘	42	13.0	21.0	62%	✔
	Papadimitriou	40.0	39.0	1.0	✘	30.0	10.0	✘	24	22.0	18.0	45%	✔
	Amirdelfan (Low)	52.1	28.3	23.9	✔	31.9	20.3	✔	36	30.7	21.4	41%	✔
	Amirdelfan (High)	50.7	31.7	19.0	✔	29.6	21.1	✔	36	25.0	25.7	51%	✔
	Lewandrowski	44.8	13.5	31.3	✔	11.8	33.0	✔	24	6.1	38.3	85%	✔
	Pang	51.0	10.0	41.0	✔	10.0	41.0	✔	24	12.5	38.5	76%	✔
Cocktail of cell types	Xu^d^	63.3	19.7	43.6	✔^d^	17.5	45.8	✔^d^	24	18.5	44.8	71%	✔^d^
	Atluri	46.1	29.9	16.2	✘	31.1	15.0	✘	12	31.1	15.0	33%	✘
	Haines	33.5	‐	‐	‐	21.0	12.5	✘	12	21.0	12.5	37%	✘
	Pettine	56.7	24.4	32.3	✔	25.0	31.7	✔	36	17.5	39.2	69%	✔
	Wolff	36.7	21.4	15.3	✘	23.9	12.8	✘	12	23.9	12.8	35%	✘
Platelet‐rich plasma products	Akeda (Non‐crossover)	35.7	‐	‐	‐	8.1	26.5	✔	12	8.1	26.5	74%	✔
	Akeda (Crossover)	34.6	‐	‐	‐	21.4	13.9	✘	12	21.4	13.9	40%	✘
	Kirchner	36.0	2.0	34.0	✔	‐	‐	‐	6	2.0	34.0	94%	✔
	Bise	29.8	‐	‐	‐	‐	‐	‐	1.5	23.0	6.8	23%	✘
	Li	76.6	14.3	62.3	✔	‐	‐	‐	6	14.3	62.3	81%	✔
	Levi	31.0	23.5	7.5	✘	‐	‐	‐	6	23.5	7.5	24%	✘
	Jain	36.7	18.6	18.1	✔	‐	‐	‐	6	18.6	18.1	49%	✔
	Kawabata	32.0	24.0	8.0	✘	‐	‐	‐	6	24.0	8.0	25%	✘
	Bhatia	49.2	‐	‐	‐	‐	‐	‐	3	29.5	19.7	40%	✔
	Demirci	63.7	32.5	31.2	✔	31.0	32.7	✔	12	31.0	32.7	51%	✔
	Karamanakos	74.0	‐	‐	‐	22.0	52.0	✔	12	22.0	52.0	70%	✔
	Wongjarupong	44.7	15.1	29.6	✔	‐	‐	‐	6	15.1	29.6	66%	✔
	Saraf	57.3	33.3	24.0	✔	‐	‐	‐	6	33.3	24.0	42%	✔
	Lam	64.0	‐	‐	‐	‐	‐	‐	1	16.7	47.3	74%	✔
	Jiang	50.9	6.7	44.3	✔	4.3	46.6	✔	12	4.3	46.6	92%	✔
	Comella	32.0	30.0	2.0	✘	‐	‐	‐	6	30.0	2.0	6%	✘

*Note*: Table of all identified studies and a summarizing statement regarding the reported ODI scores and ODI improvements at the final follow‐up time point.

Abbreviations: AC, articular cartilage‐derived chondrocytes; AD‐MSC, adipose‐derived mesenchymal stromal cells; BMA, bone marrow aspirate; BMC, bone marrow concentrate; BM‐MSC, bone marrow‐derived mesenchymal stromal cells; FU, follow‐up (in years); IVD‐C, intervertebral disc‐derived cells; LP‐PRP, leucocyte poor platelet‐rich plasma; LR‐PRP, leucocyte‐rich platelet‐rich plasma; MCID, minimally clinical important difference; MPC, mesenchymal precursor cells; NPC, nucleus pulposus cells; ODI, Oswestry disability index; PL, platelet lysate; PRP, platelet‐rich plasma; SVF, stromal vascular fraction; UC‐MSC, umbilical cord‐derived mesenchymal stromal cells.

^a^ Following recommendations of Maughan and Lewis.^58^

^b^ Estimated change as percentage from baseline with cell color representing low (red = 0%) to high (blue = 100%) reduction in ODI outcomes.

^c^ Involves cell transplantation after discectomy has already been able to provide pain relief.

^d^ Cell transplantation was performed concurrently with microdiscectomy.

### 3.4 Quality of life improvements

Studies that applied QoL assessment through SF12 or SF36 were limited; 12 trials were identified (Data S7). SF12/36 outcomes did suggest a trend of enhanced outcomes for physical components of the survey; however, often little change or even aggravation was observed in the mental components. The physical component summary score increases ranged from 15% to 278%, with a median improvement of 75%. For the mental component summary, only a median improvement of 4% (range: −8% to 266%) was reported. Here, a notable observation is the trend of general enhanced results for therapies complementing microdiscectomy.

### 3.5 Radiographic image improvements

The number of studies reporting on quantitative imaging measurements was notably small, with only 14 separate trials identified in our review (Data S8). Additionally, the types of imaging measurements were diverse and included MRI intensity values (*N* = 6), ADC (*N* = 1), disc height (*N* = 5), and Pfirrmann classifications (*N* = 9). Overall, these studies show the ability to generally limit the rate of degeneration. Specifically, when looking at the Pfirrmann classification, all the cell‐treated cohorts presented a sporadic improvement (ignoring studies involving cell injection to complement microdiscectomy), while the PRP trials showed general maintenance of Pfirrmann classification in their follow‐up. Reductions in disc height or MRI intensity were only reported in studies involving transplantation supplementing microdiscectomy or in which the entire disc was replaced by an allograft.

### 3.6 Safety assessment

Of the 63 trials, studies, and case reports mentioning adverse events, 14 studies reported the occurrence of SAE (Data S9). Specifically, 30 separate SAEs or 34 separate patients were identified in a total patient population of at least 1906 patients, equating to an SAE rate of 1.8%. It is noteworthy that the most common SAEs were related to disc (re)herniation and infectious complications. Moreover, the most serious complications were derived from case reports, specifically discussing often ill‐defined cell treatments.

## 4 DISCUSSION

### 4.1 Safety observations

Cell transplantation and PRP injection appeared to be remarkably well received. Limited SAEs are reported at a rate of 1.8%. This review does suggest a lower rate of complications for PRP products (0.5%) compared to cell injection (4.5%). Notably, the reported SAEs commonly involve events associated with disc degeneration, for example, reports of disc (re)herniation. These events could indicate treatment failure rather than a complication of the treatment. An additional repeating SAE is infectious in nature, such as bacteremia, discitis, and osteomyelitis. These SAEs highlight the risks associated with introducing foreign agents into the disc environment and with ex vivo production processes.^66^ Nonetheless, the rate of reported infectious SAEs remains low, at approximately 11 per 1906 patients (0.6%). These values are lower than those reported for spinal fusion surgery. For example, a meta‐analysis on surgical site infections after spine surgery revealed a rate of 3.1%.^67^ Moreover, our observations are similar to the findings of Jerome et al.^68^ on infectious complications following intradiscal PRP therapies, with a rate of 0.5%. This is despite a bias in our work associated with our inclusion of case reports specifically reporting on observed SAEs. Considering all these limitations and review aspects, cellular and PRP therapeutics present an overall favorable safety profile.^50^

### 4.2 Efficacy trends

**FIGURE 6:**
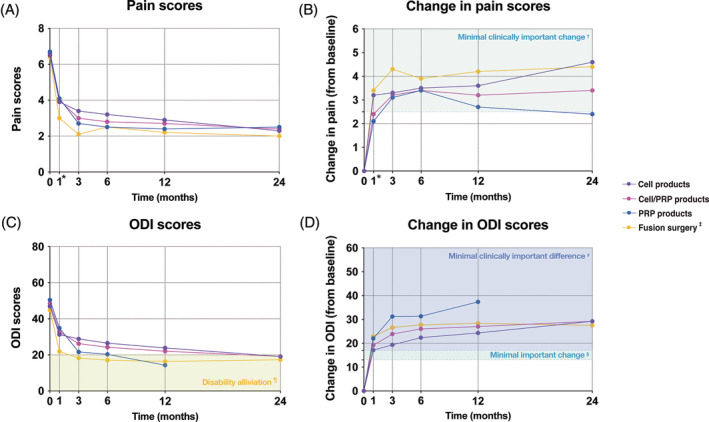
Assessment of clinical impact and comparison to spinal fusion surgery of average (A) pain scores, (B) change in pain scores from baseline, (C) average Oswestry Disability Index (ODI), (D) change in ODI from baseline presented for combined or separate cell‐ and platelet‐rich plasma (PRP) injection products. Dots represent average values recorded at indicated time point. (*) 1‐month follow‐up for cell therapy and PRP products, 1.5 months for spinal fusion. (†) Based on work of Ostelo and de Vet.^56^ (‡) Outcomes from meta‐analysis of Koenders et al.^55^ (¶) Based on the recommendations by Fairbank et al.^72^ (§) Based on the recommendations by Johnsen et al.^57^ (#) Based on the recommendations by Maughan and Lewis.^58^

The average pain and disability scores and respective reduction rates showed an evident trend of enhanced recovery for both PRP‐ and cell‐treated cohorts (Figure 6). Cell therapies might present higher rates of pain recovery and PRP‐enhanced outcomes regarding ODI scores (although the number of involved studies is limited). However, critically, these improvement rates are ideally compared to standard care. Here, we compared the trends observed here to those reported in a meta‐analysis by Koenders et al.,^55^ who analyzed pain and ODI reduction rates over 2 years in LBP patients following surgical fusion surgery. Their results are included in Figure 6 and show a decrease in LBP from 6.4 to 2.0 and a decrease in ODI from 44.8 to 17.3 over 2 years of follow‐up. Notably, the baseline and final scores appeared very similar to the data obtained from the cell‐ and PRP‐treated cohorts (Figure 6). Fusion surgery was able to engender a slightly faster pain and disability improvement (although the differences appear small; Figure 6a,c), but the long‐term outcomes are similar to trends of cell/PRP‐based therapies observed in our review. More specifically, fusion surgery did result in higher rates of pain alleviation than PRP injections but was outperformed by PRP injections when examining disability outcomes. Alternatively, cell therapy demonstrated almost identical long‐term benefits to spinal surgery in both pain and disability outcomes (Figure 6c,d). Here, it should be considered that for fusion, the painful disc is excised and fused under invasive surgery, resulting in restrictions in the patients' range of motion^69^ and risking the promotion of disc degeneration in adjacent discs.^70^, ^71^ Comparatively, cell or PRP injection involves a much less invasive procedure and is able to preserve the general disc tissue (or even restore it; Data S8), and based on current data, it is suggested to have limited associated complications, making these regenerative strategies a desirable approach. Nonetheless, careful cost‐effectiveness studies have not yet been reported and will be required to fully appreciate the potential impact of cell and PRP injections.

### 4.3 Clinical significance

Another approach to determine clinical significance is by employing “standards” indicating a minimal important change to have clinical impact, that is, MCID. In the comprehensive review of Ostelo and de Vet^56^ for both VAS and NRS pain scores, an MCID threshold of 2.5 points reduction is suggested (Figure 6b). Based on these recommendations, we illustrated a general trend to present MCID starting from 3 months, which was maintained for cell treatment for up to 2 years. Only PRP injections slightly receded below this threshold after 12 months. Similarly, MCID for ODI outcomes have been proposed; for example, Fairbank et al.^72^ had suggested an ODI of 20 to be considered successfully alleviated from disability (Figure 6c). The results from the fusion meta‐analysis discussed by Koenders et al.^55^ could reach this threshold from 3 months onwards, the cell/PRP‐treated patients in this review only approached this threshold and reached this MCID threshold at the 24‐month follow‐up. PRP was able to reach this threshold at 12 months, but no longer follow‐up data were available. Alternatively, the proposed MCID of a 17‐point change in ODI^58^ was fulfilled by both cell and PRP therapy at 1 month (Figure 6d) post‐transplantation. These observations thus highlight the ability of both PRP and cell injection to limit pain and disability outcomes in a clinically significant manner. Notably, however, the impact and significance of these MCID standards should be carefully considered. No consensus for a universal MCID exists, and it is likely to be stringently impacted by included pathologies and patient selection criteria.^73^, ^74^

### 4.4 Patient stratification

While the overall improvements suggest that cell/PRP injections are beneficial, it is important to consider the heterogeneous population reviewed here (see Data S10), including both lumbar and cervical disc injections and patients with varying conditions (e.g., herniation, radiculopathy, and spondylosis). Various therapeutic approaches are utilized, including the injection of cellular products to directly address pain and degeneration, as well as their supplementation to discectomy or even full disc replacement. As such, care should be taken when considering the averages reported in this review. It is likely that certain patient stratification will enhance the potential for pain relief or disc augmentation.^75^ For instance, maintenance of disc height, endplate integrity, and an intact AF are speculated to enhance the potential of regenerative products by enhancing the support and retention of the transplanted products.^17^, ^76^, ^77^, ^78^ Another critical restriction is the inability to link objective radiographic findings to subjective pain and disability.^79^, ^80^ This likely hinders both optimal patient stratification and outcome assessment during the trials. MRI developments aim to overcome these shortfalls but are likely still far from clinical adaptation.^79^

### 4.5 Product design and transplantation strategy

Our systematic review identified a wide range of investigated cellular and PRP‐based products with a preference for PRP products. Here, however, the radiographic data suggest that the PRP products had primarily palliative effects and showed predominant maintenance of the disc state (Data S8). In contrast, the cell‐transplanted cohorts did report sporadic disc improvement. Notably, the impact of these observations is severely limited by the small number of studies reporting quantitative MRI findings. As such, further research is needed to confirm these observations. Additionally, cell and PRP injections were most commonly applied intradiscally, despite the additional damage caused by the required disc puncture, which has been shown to promote disc degeneration.^4^, ^81^ Alternative strategies, such as epidural and intraosseous transplantation, have also been successfully applied in some of the reviewed trials and might provide interesting venues for further research.

### 4.6 Future considerations

Our review critically assessed the outcomes reported from clinical trials on cell and PRP injections for LBP and neck pain. It is important to note, however, that the studies included in our review represent only a fraction of the clinical trials registered and completed on cell therapeutics for LBP. A recent comprehensive review by Ambrosio et al. highlighted that only 26.9% of registered trials have resulted in a scientific publication.^82^ Furthermore, our analysis of the available reports revealed a generally disappointing quality in the descriptions of outcomes, study design, and statistical analyses. For instance, although many studies proposed using imaging modalities as an outcome measure, only 14 out of 68 studies (20.6%) actually included imaging‐related outcomes in their reporting. Given these findings, we urge the scientific community, including authors, peer‐reviewers, and editors, to adopt more stringent standards for the quality and transparency of reporting in human clinical trials. Additionally, we strongly recommend that the Orthopedic Research Society (ORS) Spine Section take a leading role in establishing expert consensus and designing guidelines on the expected aspects of trials and outcome measures in regenerative medicine for disc degeneration and LBP. This will aid in the formidable task of potentially translating regenerative medicine toward commercialization and into clinical practice.^66^, ^83^

### 4.7 Limitations and strengths

This review examines the use of PRP and cell‐based therapies for the alleviation of LBP and neck pain. Although the primary efficacy of PRP is attributed to the signaling factors within its enriched platelets, the final transplantation product is unlikely to be completely devoid of cells.^84^ Consequently, we considered it necessary and beneficial to include PRP in this analysis. Nevertheless, given the distinct therapeutic mechanisms speculated between PRP and cell therapies, we also aimed to carefully analyze PRP and cell injections separately. Next, this review is the first to provide a meta‐analysis of the complete collection of cell‐ and PRP‐based therapies against disc degeneration and back/neck pain. The observations and values reported in our review can be of tremendous value for future clinical trials to determine optimal cohort sizes through power analysis. Moreover, with more lenient inclusion criteria of reports, we allowed for a more holistic overview of outcomes and prevented repeated overemphasis on a small selection of randomized controlled trials. In contrast, our review is limited by the inclusion of a relatively heterogeneous patient population, treatment strategies, and outcome measures. Moreover, although we analyzed the general average outcomes per trial, the current presentation of the data was unable to consider the variability in outcomes for each included study. In addition, the included studies presented an overall high risk of bias, and many failed to clearly report on specific outcome parameters.

## 5 CONCLUSIONS

Cell and PRP transplantation for LBP and neck pain is a promising technique that could provide, for the first time, a targeted therapeutic agent to resolve the underlying cause of discogenic pain. Our review highlights the potential of these products to result in an average reduction of 3.2 points on the pain scale and 27.0 points on ODI at the 1‐year follow‐up with a generally good safety profile. These outcomes represent clinically important changes that approach similar reduction rates as patients treated with spinal fusion techniques. As such, from contemporary trials, we can conclude that cell and PRP injections are potent tools to alleviate discogenic pain. Nonetheless, further studies are needed to establish their regenerative potential and determine their cost‐effectiveness to ensure optimal allocation of health care spending.

## AUTHOR CONTRIBUTIONS

D.S. contributed as a guarantor, provided funding, and critical review of the manuscript. J.S. contributed to the design of the review, data collection, data analysis, figure production, and drafting of the manuscript. M.I. contributed as a guarantor, provided funding, and critical review of the manuscript. S.T. contributed to the design of the review, data collection, data analysis, and drafting of the manuscript. T.N.E.V. contributed to the design of the review, data collection, and critical review of the manuscript. All authors critically reviewed the work prior to submission and approved the final submitted manuscript.

## CONFLICT OF INTEREST STATEMENT

Each author certifies that D.S. has been in a paid advisory role for TUNZ Pharma Inc. (Osaka, Japan) but declares no nonfinancial competing interests. All other authors declare no financial or nonfinancial competing interests. No external funding was obtained for the production of this review. J.S. serves as a Scientific Advisor on the Editorial Board of JOR Spine, and D.S. holds the position of Editor‐in‐Chief at the same journal. Neither individual played any role nor exerted any influence in the decision‐making process concerning the publication of this article in JOR Spine.

## Supporting information


**Data S1.** Search syntax.

**Data S2.** List identified studies and involved papers for our systematic review. Trials are listed in order as presented in Table 1.

**Data S3.** Risk of bias assessment of included papers and identified trials. Assessment of risk of bias for each included reports based on the methodological index for non‐randomized studies (MINORS) scheme 1 (left) and classification through the updated method guideline for systematic reviews in the Cochrane back and neck group scheme 2 (right) for identified trials as randomized and controlled clinical trials.

**Data S4.** Tabular overview of product and transplantation method strategies of all included trials.

**Data S5.** Trends in pain alleviation following cell‐ and platelet‐rich plasma (PRP) transplantation. (A) Average pain scores and (B) average change in pain scores depicted for each identified study. Average pain scores and positive change in pain scores for cells therapies (C, D) and PRP therapies (E, F). *Sample size of <10 patients or if cohort size is unclear/unspecified. Dots represent average values recorded at indicated time point.

**Data S6.** Trends in disability alleviation following cell‐ and platelet‐rich plasma (PRP) transplantation. (A) Average Oswestry Disability Index (ODI) scores and (B) average change in ODI scores depicted for each identified study. Average ODI and change in ODI scores for cells therapies (C, D) and PRP therapies (E, F). *Sample size of <10 patients or if cohort size is unclear/unspecified. Dots represent average values recorded at indicated time point.

**Data S7.** Tabular overview of quality‐of‐life survey outcomes derived from SF36 or SF12 questionnaires.

**Data S8.** Tabular overview of radiographic reported outcomes.

**Data S9.** Tabular overview of reported serious adverse events (SAE) following cell and PRP‐transplantation for discogenic pain.

**Data S10.** Tabular overview of patient indications recruited or included into the trials and case reports.

## Data Availability

The datasets used and/or analyzed during the current study are uploaded as Supporting Information. Additional datasets are available from the corresponding author on reasonable request.
