# Supplementary material for: A comprehensive review of cell transplantation and platelet‐rich plasma therapy for the treatment of disc degeneration‐related back and neck pain: A systematic evidence‐based analysis
Source: JOR Spine. 2024 Jun 24;7(2):e1348. doi: 10.1002/jsp2.1348 (PMC11196836; doi:10.1002/jsp2.1348)
Supplement: Supplementary file 1 — Data S1. Search syntax. [file JSP2-7-e1348-s009.pdf]

**Additional file 1.| Search syntax.**

**PUBMED/MEDLINE**

*(“low back pain”[tiab] OR “intervertebral disc”[tiab] OR “IVD”[tiab] OR “disc”[tiab] OR “nucleus pulposus”[tiab] OR “annulus fibrosis”[tiab] OR “spine”[tiab] OR “spinal column”[tiab] OR “intradiscal”[tiab] OR “back pain”[tiab] OR “degenerative disc disease”[tiab] OR “endplate”[tiab] OR “disc herniation”[tiab] OR “disk herniation”[tiab] OR “intradiskal”[tiab] OR “discogenic pain”) AND (“cell therapy”[tiab] OR “cellular therapy”[tiab] OR “cell therapies”[tiab] OR “cellular therapies”[tiab] OR “cell therapeutics”[tiab] OR “cellular therapeutics”[tiab] OR “mesenchymal stem cell”[tiab] OR “stromal vascular fraction”[tiab] OR “chondrocytes”[tiab] OR “nucleus pulposus cell”[tiab] OR “stem cells”[tiab] OR “cell-based therapeutics”[tiab] OR “cell-mediated”[tiab] OR “cell transplantation”[tiab] OR “cell-based”[tiab] OR “PRP”[tiab] OR “platelet lysate”[tiab] OR “platelet-rich”[tiab] OR “platelet rich”[tiab] OR “mesenchymal precursor cells”[tiab] OR “tissue supplementation”[tiab] OR “allograft”[tiab] OR “biologics”[tiab] OR “bone marrow cells”[tiab] OR “Biologics”[tiab] OR “NOVOCART”[tiab]) AND (“clinical trial”[tiab] OR “in-human”[tiab] OR “in human”[tiab] OR “pilot study”[tiab] OR “clinic”[tiab] OR “human studies”[tiab] OR “clinical experience”[tiab] OR “clinical study”[tiab] OR “randomized controlled trial”[tiab] OR “patient”[tiab] OR “patients”[tiab] OR “trial”[tiab] OR “case presentation”[tiab] OR “case series”[tiab] OR “case report”[tiab] OR “follow-up”[tiab] OR “clinical trial”[tiab] OR “follow up”[tiab])*

**SCOPUS**

*TITLE-ABS-KEY (“low back pain” OR “intervertebral disc” OR “IVD” OR “disc” OR “nucleus pulposus” OR “annulus fibrosis” OR “spine” OR “spinal column” OR “intradiscal” OR “back pain” OR “degenerative disc disease” OR “endplate” OR “disc herniation” OR “disk herniation” OR “intradiskal” OR “discogenic pain”) AND TITLE-ABS-KEY (“cell therapy” OR “cellular therapy” OR “cell therapies” OR “cellular therapies” OR “cell therapeutics” OR “cellular therapeutics” OR “mesenchymal stem cell” OR “stromal vascular fraction” OR “chondrocytes” OR “nucleus pulposus cell” OR “stem cells” OR “cell-based therapeutics” OR “cell-mediated” OR “cell transplantation” OR “cell-based” OR “PRP” OR “platelet lysate” OR “platelet-rich” OR “platelet rich” OR “mesenchymal precursor cells” OR “tissue supplementation” OR “allograft” OR “biologics” OR “bone marrow cells” OR “Biologics” OR “NOVOCART”) AND TITLE-ABS-KEY (“clinical trial” OR “in-human” OR “in human” OR “pilot study” OR “clinic” OR “human studies” OR “clinical experience” OR “clinical study” OR “randomized controlled trial” OR “patient” OR “patients” OR “trial” OR “case presentation” OR “case series” OR “case report” OR “follow-up” OR “clinical trial” OR “follow up”)*

**WEB OF SCIENCE**

*TS (“low back pain” OR “intervertebral disc” OR “IVD” OR “disc” OR “nucleus pulposus” OR “annulus fibrosis” OR “spine” OR “spinal column” OR “intradiscal” OR “back pain” OR “degenerative disc disease” OR “endplate” OR “disc herniation” OR “disk herniation” OR “intradiskal” OR “discogenic pain” OR “intervertebral disk” OR “IVD” OR “disk” OR “intradiskal” OR “back pain” OR “degenerative disk disease” OR “disk herniation” OR “diskogenic pain” OR “low-back-pain” OR “intervertebral-disc” OR “nucleus-pulposus” OR “annulus-fibrosis” OR “spinal-column” OR “intradiscal” OR “back-pain” OR “degenerative-disc” OR “endplate” OR “disc-herniation” OR “disk-herniation” OR “intradiskal” OR “discogenic-pain” OR “intervertebral-disk” OR “IVD” OR “disk” OR “back-pain” OR “degenerative-disk” OR “disk-herniation” OR “discogenic-pain”) AND TS (“cell therapy” OR “cellular therapy” OR “cell therapies” OR “cellular therapies” OR “cell therapeutics” OR “cellular therapeutics” OR “mesenchymal stromal cell” OR “mesenchymal stem cell” OR “stromal vascular fraction” OR “chondrocytes” OR “nucleus pulposus cell” OR “stem cell” OR “cell-based therapeutics” OR “cell-mediated” OR “cell*

*transplantation" OR "cell-based" OR "PRP" OR "platelet lysate" OR "platelet-rich" OR "platelet rich" OR "mesenchymal precursor cells" OR "tissue supplementation" OR "allograft" OR "biologics" OR "bone marrow cells" OR "Biologics" OR "NOVOCART" OR "cell-therapy" OR "cellular-therapy" OR "cell-therapies" OR "cellular-therapies" OR "cell-therapeutics" OR "cellular-therapeutics" OR "stem-cell" OR "cell-transplantation" OR "PRP" OR "platelet-lysate" OR "tissue-supplementation") AND TS ("clinical trial" OR "in-human" OR "in human" OR "pilot study" OR "clinic" OR "human studies" OR "clinical experience" OR "clinical study" OR "randomized controlled trial" OR "patient" OR "patients" OR "trial" OR "case presentation" OR "case series" OR "case report" OR "follow-up" OR "clinical trial" OR "follow up" OR "clinical-trial" OR "pilot-study" OR "clinical-study" OR "case-presentation" OR "case-series" OR "case-report" OR "follow-up")*
