# Supplementary material for: A comprehensive review of cell transplantation and platelet‐rich plasma therapy for the treatment of disc degeneration‐related back and neck pain: A systematic evidence‐based analysis
Source: JOR Spine. 2024 Jun 24;7(2):e1348. doi: 10.1002/jsp2.1348 (PMC11196836; doi:10.1002/jsp2.1348)
Supplement: Supplementary file 2 — Data S2. List identified studies and involved papers for our systematic review. Trials are listed in order as presented in Table 1. [file JSP2-7-e1348-s008.pdf]

Additional file 2.I List identified studies and involved papers for our systematic review. Trials are listed in order as presented in table 1.

Chondrogenic cells

Coric

Coric D, Pettine K, Sumich A, Boltes MO. Prospective study of disc repair with allogeneic chondrocytes presented at the 2012 Joint Spine Section Meeting. *Journal of neurosurgery Spine.* 2013;18(1):85-95.

Ruan

Ruan D, He Q, Ding Y, Hou L, Li J, Luk KD. Intervertebral disc transplantation in the treatment of degenerative spine disease: a preliminary study. *Lancet (London, England).* 2007;369(9566):993-999.

Zhang

Zhang J, Ruan D, Xuan A, et al. Comparative study of outcomes between allograft intervertebral disc transplantation and anterior cervical discectomy and fusion: a retrospective cohort study at least 5 years of follow-up. *Eur Spine J.* 2023.

Meisel

Meisel HJ, Ganey T, Hutton WC, Libera J, Minkus Y, Alasevic O. Clinical experience in cell-based therapeutics: intervention and outcome. *Eur Spine J.* 2006;15 Suppl 3(Suppl 3):S397-405.

Meisel HJ, Siodla V, Ganey T, Minkus Y, Hutton WC, Alasevic OJ. Clinical experience in cell-based therapeutics: disc chondrocyte transplantation A treatment for degenerated or damaged intervertebral disc. *Biomolecular engineering.* 2007;24(1):5-21.

Tschugg

Tschugg A, Diepers M, Simone S, et al. A prospective randomized multicenter phase I/II clinical trial to evaluate safety and efficacy of NOVOCART disk plus autologous disk chondrocyte transplantation in the treatment of nucleotomized and degenerative lumbar disks to avoid secondary disease: safety results of Phase I-a short report. *Neurosurgical review.* 2017;40(1):155-162.

Schwan

Schwan S, Ludtka C, Friedmann A, et al. Calcium Microcrystal Formation in Recurrent Herniation Patients After Autologous Disc Cell Transplantation. *Tissue Eng Regen Med.* 2017;14(6):803-814.

Xuan

Xuan A, Ruan D, Wang C, et al. Intradiscal Injection of Autologous Discogenic Cells in Patients with Discectomy: A Prospective Clinical Study of Its Safety and Feasibility. *Stem cells translational medicine.* 2022;11(5):490-503.

Mochida

Mochida J, Sakai D, Nakamura Y, Watanabe T, Yamamoto Y, Kato S. Intervertebral disc repair with activated nucleus pulposus cell transplantation: a three-year, prospective clinical study of its safety. *European cells & materials.* 2015;29:202-212; discussion 212.

Hunter

Hunter CW, Guyer R, Froimson M, DePalma MJ. Effect of age on outcomes after allogeneic disc tissue supplementation in patients with chronic discogenic low back pain in the VAST trial. *Pain Manag.* 2022;12(3):301-311.

Beall DP, Davis T, DePalma MJ, et al. Viable Disc Tissue Allograft Supplementation; One- and Two-level Treatment of Degenerated Intervertebral Discs in Patients with Chronic Discogenic Low Back Pain: One Year Results of the VAST Randomized Controlled Trial. *Pain physician.* 2021;24(6):465-477.

Beall DP, Wilson GL, Bishop R, Tally W. VAST Clinical Trial: Safely Supplementing Tissue Lost to Degenerative Disc Disease. *International journal of spine surgery.* 2020;14(2):239-253.

Mesenchymal stromal cells

Jung

Jung JW, Kwon M, Choi JC, et al. Familial occurrence of pulmonary embolism after intravenous, adipose tissue-derived stem cell therapy. *Yonsei medical journal.* 2013;54(5):1293-1296.

Piccirilli

Piccirilli M, Delfinis CP, Santoro A, Salvati M. Mesenchymal stem cells in lumbar spine surgery: a single institution experience about red bone marrow and fat tissue derived MSCs. *Journal of neurosurgical sciences.* 2017;61(2):124-133.

Kumar

Kumar H, Ha DH, Lee EJ, et al. Safety and tolerability of intradiscal implantation of combined autologous adipose-derived mesenchymal stem cells and hyaluronic acid in patients with chronic discogenic low back pain: 1-year follow-up of a phase I study. *Stem cell research & therapy.* 2017;8(1):262.

Bates

Bates D, Vivian D, Freitag J, et al. Low-dose mesenchymal stem cell therapy for discogenic pain: safety and efficacy results from a 1-year feasibility study. *Future Sci OA.* 2022;8(5):FSO794.

Orozco

Orozco L, Soler R, Morera C, Alberca M, Sanchez A, Garcia-Sancho J. Intervertebral disc repair by autologous mesenchymal bone marrow cells: a pilot study. *Transplantation.* 2011;92(7):822-828.

Noriega

Garcia-Sancho J, Sanchez A, Vega A, Noriega DC, Nocito M. Influence of HLA Matching on the Efficacy of Allogeneic Mesenchymal Stromal Cell Therapies for Osteoarthritis and Degenerative Disc Disease. *Transplant Direct.* 2017;3(9):e205.

Noriega DC, Ardura F, Hernandez-Ramajo R, et al. Treatment of Degenerative Disc Disease With Allogeneic Mesenchymal Stem Cells: Long-term Follow-up Results. *Transplantation.* 2021;105(2):e25-e27.

Noriega DC, Ardura F, Hernandez-Ramajo R, et al. Intervertebral Disc Repair by Allogeneic Mesenchymal Bone Marrow Cells: A Randomized Controlled Trial. *Transplantation.* 2017;101(8):1945-1951.

Papadimitriou

Papadimitriou N, Hebelka H, Hingert D, et al. Intradiscal Injection of Iron-Labeled Autologous Mesenchymal Stromal Cells in Patients With Chronic Low Back Pain: A Feasibility Study With 2 Years Follow-Up. *International journal of spine surgery.* 2021;15(6):1201-1209.

Amirdelfan

Amirdelfan K, Bae H, McJunkin T, et al. Allogeneic mesenchymal precursor cells treatment for chronic low back pain associated with degenerative disc disease: a prospective randomized, placebo-controlled 36-month study of safety and efficacy. *The spine journal : official journal of the North American Spine Society.* 2021;21(2):212-230.

Ju DG, Kanim LE, Bae HW. Is There Clinical Improvement Associated With Intradiscal Therapies? A Comparison Across Randomized Controlled Studies. *Global spine journal.* 2022;12(5):756-764.

Lewandrowski

Lewandrowski KU, Dowling A, Vera JC, Leon JFR, Telfeian AE, Lorio MP. Pain Relief After Allogenic Stem Cell Disc Therapy. *Pain physician.* 2023;26(2):197-206.

Pang

Pang X, Yang H, Peng B. Human umbilical cord mesenchymal stem cell transplantation for the treatment of chronic discogenic low back pain. *Pain physician.* 2014;17(4):E525-530.

Cocktail

Xu

Xu B, Zhang H, Du L, et al. Selective Retention of Bone Marrow Stromal Cells with Gelatin Sponge for Repair of Intervertebral Disc Defects after Microendoscopic Discectomy: A Prospective Controlled Study and 2-Year Follow-Up. *BioMed research international.* 2021;2021:4822383.

Atluri

Atluri S, Murphy MB, Dragella R, et al. Evaluation of the Effectiveness of Autologous Bone Marrow Mesenchymal Stem Cells in the Treatment of Chronic Low Back Pain Due to Severe Lumbar Spinal Degeneration: A 12-Month, Open-Label, Prospective Controlled Trial. *Pain physician.* 2022;25(2):193-207.

Haines

Haines CM, Bhatt FR, Orosz LD, et al. Low Back Pain, Disability, and Quality of Life One Year following Intradiscal Injection of Autologous Bone Marrow Aspirate Concentrate. *Stem Cells Int.* 2022;2022:9617511.

Pettine

Pettine K, Suzuki R, Sand T, Murphy M. Treatment of discogenic back pain with autologous bone marrow concentrate injection with minimum two year follow-up. *Int Orthop.* 2016;40(1):135-140.

Pettine KA, Murphy MB, Suzuki RK, Sand TT. Percutaneous injection of autologous bone marrow concentrate cells significantly reduces lumbar discogenic pain through 12 months. *Stem cells (Dayton, Ohio).* 2015;33(1):146-156.

Pettine KA, Suzuki RK, Sand TT, Murphy MB. Autologous bone marrow concentrate intradiscal injection for the treatment of degenerative disc disease with three-year follow-up. *Int Orthop.* 2017;41(10):2097-2103.

Wolff

Wolff M, Shillington JM, Rathbone C, Piasecki SK, Barnes B. Injections of concentrated bone marrow aspirate as treatment for Discogenic pain: a retrospective analysis. *BMC musculoskeletal disorders.* 2020;21(1):135.

El-Kadiry

El-Kadiry AE, Lumbao C, Rafei M, Shammaa R. Autologous BMAC Therapy Improves Spinal Degenerative Joint Disease in Lower Back Pain Patients. *Front Med (Lausanne).* 2021;8:622573.

Jerome

Jerome MA, Lutz C, Lutz GE. Risks of Intradiscal Orthobiologic Injections: A Review of the Literature and Case Series Presentation. *International journal of spine surgery.* 2021;15(s1):26-39.

Platelet-derived plasma product

Centeno

Centeno C, Markle J, Dodson E, et al. The use of lumbar epidural injection of platelet lysate for treatment of radicular pain. *J Exp Orthop.* 2017;4(1):38.

Akeda

Akeda K, Ohishi K, Takegami N, et al. Platelet-Rich Plasma Releasate versus Corticosteroid for the Treatment of Discogenic Low Back Pain: A Double-Blind Randomized Controlled Trial. *J Clin Med.* 2022;11(2).

Akeda K, Fujiwara T, Takegami N, Yamada J, Sudo A. Retrospective Analysis of Factors Associated with the Treatment Outcomes of Intradiscal Platelet-Rich Plasma-Releasate Injection Therapy for Patients with Discogenic Low Back Pain. *Medicina (Kaunas).* 2023;59(4).

Akeda

Akeda K, Ohishi K, Masuda K, et al. Intradiscal Injection of Autologous Platelet-Rich Plasma Releasate to Treat Discogenic Low Back Pain: A Preliminary Clinical Trial. *Asian Spine J.* 2017;11(3):380-389.

Akeda K, Takegami N, Yamada J, et al. Platelet-Rich Plasma-Releasate (PRPr) for the Treatment of Discogenic Low Back Pain Patients: Long-Term Follow-Up Survey. *Medicina (Kaunas).* 2022;58(3).

Kirchner

Kirchner F, Anitua E. Minimally Invasive PRGF Treatment for Low Back Pain and Degenerative Disc Disease. In: Anitua E, Cugat R, Sánchez M, eds. *Platelet Rich Plasma in Orthopaedics and Sports Medicine.* Cham: Springer International Publishing; 2018:259-275.

Kirchner

Kirchner F, Anitua E. Intradiscal and intra-articular facet infiltrations with plasma rich in growth factors reduce pain in patients with chronic low back pain. *J Craniovertebr Junction Spine.* 2016;7(4):250-256.

Kirchner

Kirchner F, Pinar A, Milani I, Prado R, Padilla S, Anitua E. Vertebral intraosseous plasma rich in growth factor (PRGF-Endoret) infiltrations as a novel strategy for the treatment of degenerative lesions of endplate in lumbar pathology: description of technique and case presentation. *J Orthop Surg Res.* 2020;15(1):72.

Beatty

Beatty NR, Lutz C, Boachie-Adjei K, Leynes TA, Lutz C, Lutz G. Spondylodiscitis due to Cutibacterium acnes following lumbosacral intradiscal biologic therapy: a case report. *Regen Med.* 2019;14(9):823-829.

Bise

Bise S, Dallaudiere B, Pesquer L, et al. Comparison of interlaminar CT-guided epidural platelet-rich plasma versus steroid injection in patients with lumbar radicular pain. *Eur Radiol.* 2020;30(6):3152-3160.

Kirchner

Kirchner F, Milani I, Martinez A, et al. Plasma Rich in Growth Factors (PRGF) in the Treatment of Cervical and Lumbar Back Pain: A Retrospective Observational Clinical Study. *Pain physician.* 2021;24(5):E649-E660.

Zielinski

Zielinski MA, Evans NE, Bae H, et al. Safety and Efficacy of Platelet Rich Plasma for Treatment of Lumbar Discogenic Pain: A Prospective, Multicenter, Randomized, Double-blind Study. *Pain physician.* 2022;25(1):29-34.

Li

Li J, Yuan X, Li F, et al. A novel full endoscopic annular repair technique combined with autologous conditioned plasma intradiscal injection: a new safe serial therapeutic model for the treatment of lumbar disc herniation. *Ann Palliat Med.* 2021;10(1):292-301.

Zhang

Zhang J, Liu D, Gong Q, Chen J, Wan L. Intradiscal Autologous Platelet-Rich Plasma Injection for Discogenic Low Back Pain: A Clinical Trial. *BioMed research international.* 2022;2022:9563693.

Lam

Lam KHS, Hung CY, Wu TJ, et al. Novel Ultrasound-Guided Cervical Intervertebral Disc Injection of Platelet-Rich Plasma for Cervicodiscogenic Pain: A Case Report and Technical Note. *Healthcare (Basel).* 2022;10(8).

Le

Le VT, Nguyen Dao LT, Nguyen AM. Transforaminal injection of autologous platelet-rich plasma for lumbar disc herniation: A single-center prospective study in Vietnam. *Asian J Surg.* 2023;46(1):438-443.

Levi

Levi D, Horn S, Tyszko S, Levin J, Hecht-Leavitt C, Walko E. Intradiscal Platelet-Rich Plasma Injection for Chronic Discogenic Low Back Pain: Preliminary Results from a Prospective Trial. *Pain medicine (Malden, Mass).* 2016;17(6):1010-1022.

Cheng

Cheng J, Santiago KA, Nguyen JT, Solomon JL, Lutz GE. Treatment of symptomatic degenerative intervertebral discs with autologous platelet-rich plasma: follow-up at 5-9 years. *Regen Med.* 2019;14(9):831-840.

Ruiz-Lopez

Ruiz-Lopez R, Tsai YC. A Randomized Double-Blind Controlled Pilot Study Comparing Leucocyte-Rich Platelet-Rich Plasma and Corticosteroid in Caudal Epidural Injection for Complex Chronic Degenerative Spinal Pain. *Pain practice : the official journal of World Institute of Pain.* 2020;20(6):639-646.

Jain

Jain D, Goyal T, Verma N, Paswan AK, Dubey RK. Intradiscal Platelet-Rich Plasma Injection for Discogenic Low Back Pain and Correlation with Platelet Concentration: A Prospective Clinical Trial. *Pain medicine (Malden, Mass).* 2020;21(11):2719-2725.

Lam

Lam KHS, Hung CY, Wu TJ. Ultrasound-guided L5/S1 intradiscal needle placement using biplanar approach with the patient in the lateral decubitus position: A report of three cases. *Pain practice : the official journal of World Institute of Pain.* 2022;22(1):117-122.

Kawabata

Kawabata S, Hachiya K, Nagai S, et al. Autologous Platelet-Rich Plasma Administration on the Intervertebral Disc in Low Back Pain Patients with Modic Type 1 Change: Report of Two Cases. *Medicina (Kaunas).* 2023;59(1).

Monfett

Monfett M, Harrison J, Boachie-Adjei K, Lutz G. Intradiscal platelet-rich plasma (PRP) injections for discogenic low back pain: an update. *Int Orthop.* 2016;40(6):1321-1328.

Tuakli-Wosornu YA, Terry A, Boachie-Adjei K, et al. Lumbar Intradiskal Platelet-Rich Plasma (PRP) Injections: A Prospective, Double-Blind, Randomized Controlled Study. *PM & R : the journal of injury, function, and rehabilitation.* 2016;8(1):1-10; quiz 10.

Bhatia

Bhatia R, Chopra G. Efficacy of Platelet Rich Plasma via Lumbar Epidural Route in Chronic Prolapsed Intervertebral Disc Patients-A Pilot Study. *J Clin Diagn Res.* 2016;10(9):UC05-UC07.

Demirci

Demirci AY. The retrospective analysis of platelet-rich plasma and corticosteroid injection under epiduroscopic guidance for radiculopathy in operated or unoperated patients for lumbar disc herniation. *Turk J Phys Med Rehabil.* 2022;68(3):409-417.

Navani

Navani A, Hames A. Platelet-rich plasma injections for lumbar discogenic pain: A preliminary assessment of structural and functional changes. *Techniques in Regional Anesthesia and Pain Management.* 2015;19(1-2):38-44.

Karamanakos

Karamanakos PN, Manousakis E, Rozakis D, Kamarainen OP, Oikonomi E, Panteli ES. Intradiscal Platelet-Rich Plasma for Discogenic Low Back Pain Owing to a Degenerated and Previously Discectomized L5-S1 Disc. *Pain medicine (Malden, Mass).* 2021;22(5):1235-1236.

Singh

Singh GK, Talawar P, Kumar A, Sharma RS, Purohit G, Bhandari B. Effect of autologous platelet-rich plasma (PRP) on low back pain in patients with prolapsed intervertebral disc: A randomised controlled trial. *Indian J Anaesth.* 2023;67(3):277-282.

Wongjarupong

Wongjarupong A, Pairuchvej S, Laohapornsvan P, et al. "Platelet-Rich Plasma" epidural injection an emerging strategy in lumbar disc herniation: a Randomized Controlled Trial. *BMC musculoskeletal disorders.* 2023;24(1):335.

Saraf

Saraf A, Hussain A, Sandhu AS, Bishnoi S, Arora V. Transforaminal Injections of Platelet-Rich Plasma Compared with Steroid in Lumbar radiculopathy: A Prospective, Double-Blind Randomized Study. *Indian J Orthop.* 2023;57(7):1126-1133.

Lutz

Lutz GE. Increased Nuclear T2 Signal Intensity and Improved Function and Pain in a Patient One Year After an Intradiscal Platelet-Rich Plasma Injection. *Pain medicine (Malden, Mass).* 2017;18(6):1197-1199.

Lam

Lam KHS, Hung CY, Wu TJ. Ultrasound-Guided Cervical Intradiscal Injection with Platelet-Rich Plasma with Fluoroscopic Validation for the Treatment of Cervical Discogenic Pain: A Case Presentation and Technical Illustration. *Journal of pain research.* 2020;13:2125-2129.

Wu

Wu TJ, Hung CY, Lee CW, Lam S, Clark TB, Chang KV. Ultrasound-Guided Lumbar Intradiscal Injection for Discogenic Pain: Technical Innovation and Presentation of Two Cases. *Journal of pain research.* 2020;13:1103-1107.

Xu

Xu Z, Wu S, Li X, Liu C, Fan S, Ma C. Ultrasound-Guided Transforaminal Injections of Platelet-Rich Plasma Compared with Steroid in Lumbar Disc Herniation: A Prospective, Randomized, Controlled Study. *Neural Plast.* 2021;2021:5558138.

Jiang

Jiang Y, Zuo R, Yuan S, et al. Transforaminal Endoscopic Lumbar Discectomy with versus without Platelet-Rich Plasma Injection for Lumbar Disc Herniation: A Prospective Cohort Study. *Pain Res Manag.* 2022;2022:6181478.

Godek

Godek P. High Volume PRP Therapy. *Ortop Traumatol Rehabil.* 2022;24(1):43-60.

Lutz

Lutz C, Cheng J, Prysak M, Zukofsky T, Rothman R, Lutz G. Clinical outcomes following intradiscal injections of higher-concentration platelet-rich plasma in patients with chronic lumbar discogenic pain. *Int Orthop.* 2022;46(6):1381-1385.

Rawson

Rawson B. Platelet-Rich Plasma and Epidural Platelet Lysate: Novel Treatment for Lumbar Disk Herniation. *J Am Osteopath Assoc.* 2020;120(3):201-207.

Williams

Williams C, Jerome M, Fausel C, Dodson E, Stemper I, Centeno C. Regenerative Injection Treatments Utilizing Platelet Products and Prolotherapy for Cervical Spine Pain: A Functional Spinal Unit Approach. *Cureus.* 2021;13(10):e18608.

Other

Subach

Subach BR, Copay AG, Martin MM, Schuler TC, DeWolfe DS. Epidural abscess and cauda equina syndrome after percutaneous intradiscal therapy in degenerative lumbar disc disease. *The spine journal : official journal of the North American Spine Society.* 2012;12(11):e1-4.

Centeno

Elabd C, Centeno CJ, Schultz JR, Lutz G, Ichim T, Silva FJ. Intra-discal injection of autologous, hypoxic cultured bone marrow-derived mesenchymal stem cells in five patients with chronic lower back pain: a long-term safety and feasibility study. *J Transl Med.* 2016;14(1):253.

Centeno C, Markle J, Dodson E, et al. Treatment of lumbar degenerative disc disease-associated radicular pain with culture-expanded autologous mesenchymal stem cells: a pilot study on safety and efficacy. *J Transl Med.* 2017;15(1):197.

Ramos

Ramos O, Speirs JN, Danisa O. Lumbar Discitis and Osteomyelitis After a Spinal Stem Cell Injection?: A Case Report and Literature Review. *JBJS Case Connect.* 2020;10(3):e1900636.

Comella

Comella K, Silbert R, Parlo M. Effects of the intradiscal implantation of stromal vascular fraction plus platelet rich plasma in patients with degenerative disc disease. *J Transl Med.* 2017;15(1):12.

Singh

Singh V, McGuffin S, Sabo M, Nicholls M. Lumbar Spine Osteomyelitis and Diskitis following Intradiskal Stem Cell Injections. *PM & R : the journal of injury, function, and rehabilitation.* 2020;12(6):624-625.
