# Supplementary material for: A comprehensive review of cell transplantation and platelet‐rich plasma therapy for the treatment of disc degeneration‐related back and neck pain: A systematic evidence‐based analysis
Source: JOR Spine. 2024 Jun 24;7(2):e1348. doi: 10.1002/jsp2.1348 (PMC11196836; doi:10.1002/jsp2.1348)
Supplement: Supplementary file 3 — Data S3. Risk of bias assessment of included papers and identified trials. Assessment of risk of bias for each included reports based on the methodological index for non‐randomized studies (MINORS) scheme 1 (left) and classification through the updated method guideline for systematic reviews in the Cochrane back and neck group scheme 2 (right) for identified trials as randomized and controlled clinical trials. [file JSP2-7-e1348-s002.pdf]

Additional file 3.I Risk of bias assessment of included papers and identified trials. Assessment of risk of bias for each included reports based on the Methodological index for non-randomized studies (MINORS) scheme[1] (Left) and classification through the Updated Method Guideline for Systematic Reviews in the Cochrane Back and Neck Group scheme[2] (Right) for identified trials as randomized and controlled clinical trials.

No	Author	Year	Clearly stated aim	Inclusion of consecutive patients	Prospective Data Collection	Endpoints Appropriate to Study aims	Unbiased Assessment of Study Endpoints	Follow-up Period Appropriate to Study Aim	<5% Lost to Follow-up	Propspective Calculation of Study Size	Adequate Control group	Contemporary Groups	Baseline Equivalence of Groups	Adequate Statiscal Analysis	Sum score
1	Coric	2013													10
2	Meisel	2006													14
3	Meisel	2007													14
4	Tschugg	2017													12
5	Schwan	2017													7
6	Xuan	2022													18
7	Mochida	2015													13
8	Hunter	2022													16
9	Beall	2021													18
10	Beall	2020													17
11	Jung	2013													4
12	Piccirilli	2017													6
13	Kumar	2017													12
14	Orozco	2011													11
15	Gacia-Sancho	2011													3
16	Noriega	2017													17
17	Noriega	2017													13
18	Papadimitriou	2021													9
19	Amirdelfan	2021													19
20	Ju	2022													14
21	Pang	2014													8
22	Xu	2021													19
23	Pettine	2016													10
24	Pettine	2015													10
25	Pettine	2017													10
26	Wolff	2020													6
27	El-Kadiry	2021													10
28	Jerome	2021													6
29	Centeno	2017													5
30	Kirchner	2016													5
31	Kirchner	2020													3
32	Levi	2016													8
33	Monfett	2016													7
34	Tuakli-Wosornu	2016													18
35	Bhatia	2016													10
36	Lutz	2017													5
37	Beatty	2019													4
38	Cheng	2019													10
39	Lam	2020													3
40	Wu	2020													3
41	Bise	2020													19
42	Ruiz-Lopez	2020													20
43	Jain	2020													10
44	Xu	2021													22
45	Kirchner	2021													6
46	Zielinski	2022													13
47	Jiang	2022													19
48	Lam	2022													2
49	Godek	2022													3
50	Le	2022													10
51	Lutz	2022													9
52	Akeda	2022													22
53	Akeda	2017													11
54	Akeda	2022													9
55	Subach	2012													6
56	Elabd	2016													10
57	Centeno	2017													8
58	Ramos	2020													5
59	Rawson	2020													4
60	Comella	2017													10

POINTS	
0	Not reported
1	Reported but inadequate
2	Reported and adequate

Non-controled studies

Comparative studies

No	Study ID	Was method of randomization adequate	Was treatment allocation concealed	Was patient blind to the intervention	Was care provider blinded to intervention	Was outcome assessor blinded to intervention	Was drop-out rate described and acceptable	Were all randomized participant analyzed in group of allocation	Are reports of study free of suggestion of selective outcome reporting	Were groups similar at baseline regarding most important progn. indicators	Were cointerventions avoided or similar	Was the compliance acceptable in all groups	Was the timing of the outcome similar in all groups	Are there (likely) other sources of potential bias
1	Meisel													
2	Tschugg													
3	Hunter													
4	Noriega													
5	Ju													
6	Xu													
7	Tuakli-Wosornu													
8	Ruiz-Lopez													
9	Xu													
10	Zielinski													
11	Akeda													

POINTS
No
Unsure
Yes

Additional file to "A Comprehensive Review of Cell Transplantation and Platelet Rich Plasma Therapy for the Treatment of Disc Degeneration-Related Back and Neck Pain: A Systematic Evidence-Based Analysis" by J Schol, S Tamagawa, et al. (2024) JOR Spine

REFERENCES

1. Slim K, Nini E, Forestier D, Kwiatkowski F, Panis Y, Chipponi J. Methodological index for non-randomized studies (minors): development and validation of a new instrument. *ANZ J Surg.* 2003;73(9):712-716.
2. Furlan AD, Malmivaara A, Chou R, et al. 2015 Updated Method Guideline for Systematic Reviews in the Cochrane Back and Neck Group. *Spine (Phila Pa 1976).* 2015;40(21):1660-1673.
