# Supplementary material for: A comprehensive review of cell transplantation and platelet‐rich plasma therapy for the treatment of disc degeneration‐related back and neck pain: A systematic evidence‐based analysis
Source: JOR Spine. 2024 Jun 24;7(2):e1348. doi: 10.1002/jsp2.1348 (PMC11196836; doi:10.1002/jsp2.1348)
Supplement: Supplementary file 5 — Data S5. Trends in pain alleviation following cell‐ and platelet‐rich plasma (PRP) transplantation. (A) Average pain scores and (B) average change in pain scores depicted for each identified study. Average pain scores and positive change in pain scores for cells therapies (C, D) and PRP therapies (E, F). *Sample size of <10 patients or if cohort size is unclear/unspecified. Dots represent average values recorded at indicated time point. [file JSP2-7-e1348-s001.pdf]

Additional file 5.I Trends in pain alleviation following cell and platelet rich plasma (PRP) transplantation. (**a**) Average pain scores and (**b**) average change in pain scores depicted for each identified study. Average pain scores and positive change in pain scores for cells therapies (**c, d**) and PRP therapies (**e, f**). *Sample size of <10 patients or if cohort size is unclear/unspecified. Dots represent average values recorded at indicated time point.

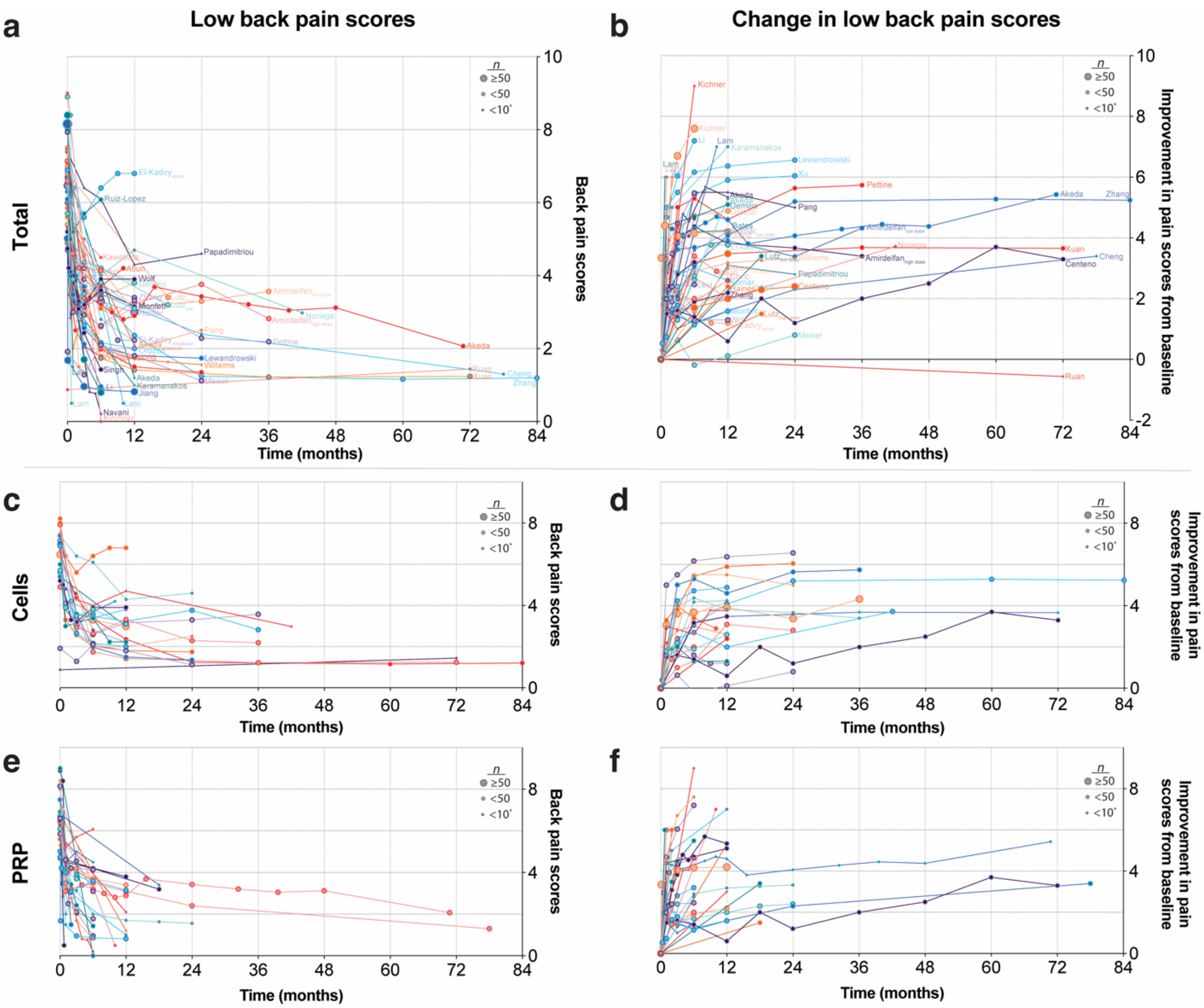

Additional file to "A Comprehensive Review of Cell Transplantation and Platelet Rich Plasma Therapy for the Treatment of Disc Degeneration-Related Back and Neck Pain: A Systematic Evidence-Based Analysis" by J Schol, S Tamagawa, et al. (2024) JOR Spine
