# Supplementary material for: A comprehensive review of cell transplantation and platelet‐rich plasma therapy for the treatment of disc degeneration‐related back and neck pain: A systematic evidence‐based analysis
Source: JOR Spine. 2024 Jun 24;7(2):e1348. doi: 10.1002/jsp2.1348 (PMC11196836; doi:10.1002/jsp2.1348)

Additional file 6.I Trends in disability alleviation following cell and platelet rich plasma (PRP) transplantation. (**a**) Average Oswestry Disability Index (ODI) scores and (**b**) average change in ODI scores depicted for each identified study. Average ODI and change in ODI scores for cells therapies (**c, d**) and PRP therapies (**e, f**). *Sample size of <10 patients or if cohort size is unclear/unspecified. Dots represent average values recorded at indicated time point.

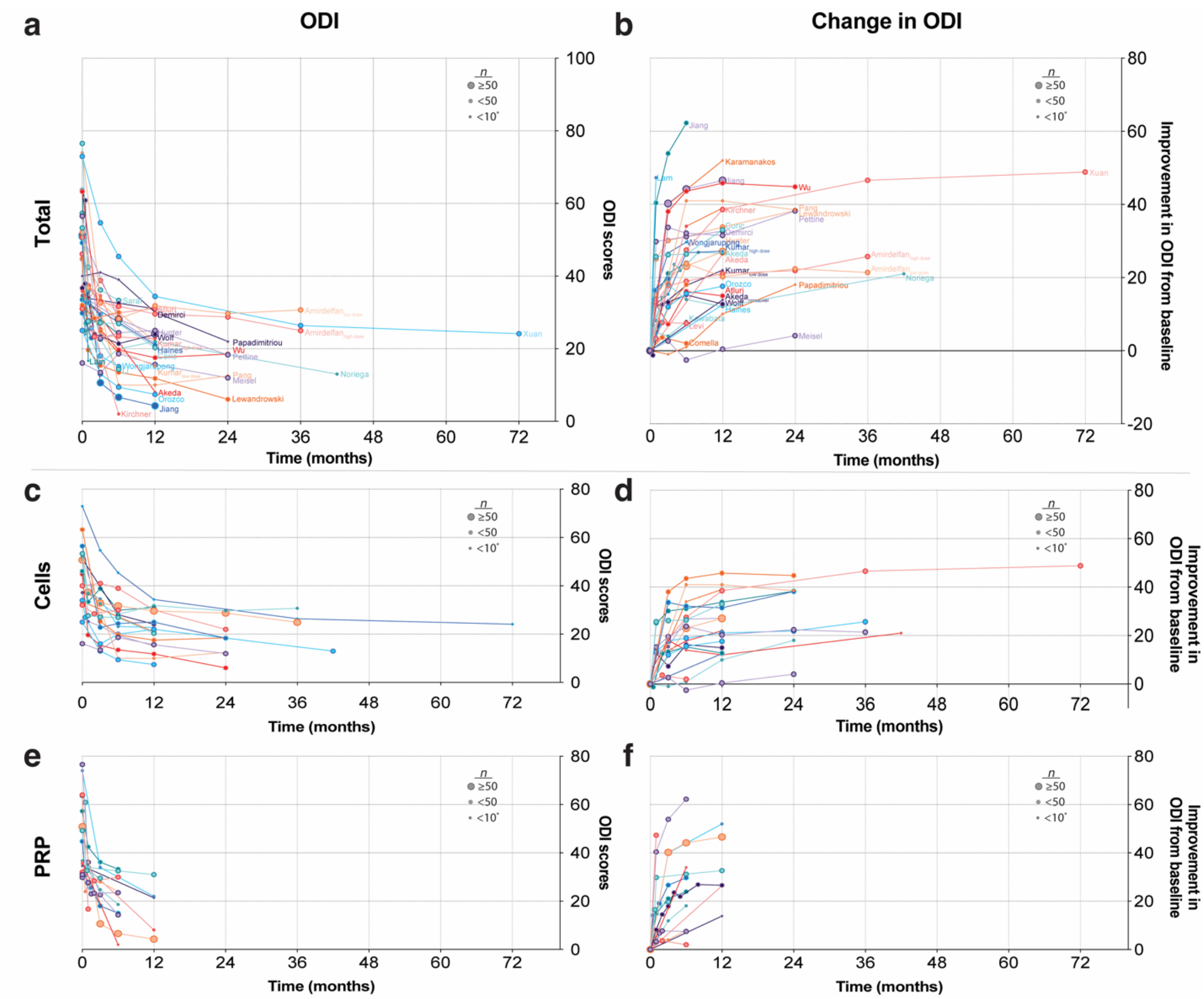

Supplement: Supplementary file 6 — Data S6. Trends in disability alleviation following cell‐ and platelet‐rich plasma (PRP) transplantation. (A) Average Oswestry Disability Index (ODI) scores and (B) average change in ODI scores depicted for each identified study. Average ODI and change in ODI scores for cells therapies (C, D) and PRP therapies (E, F). *Sample size of <10 patients or if cohort size is unclear/unspecified. Dots represent average values recorded at indicated time point. [file JSP2-7-e1348-s005.pdf]
