# Supplementary material for: A comprehensive review of cell transplantation and platelet‐rich plasma therapy for the treatment of disc degeneration‐related back and neck pain: A systematic evidence‐based analysis
Source: JOR Spine. 2024 Jun 24;7(2):e1348. doi: 10.1002/jsp2.1348 (PMC11196836; doi:10.1002/jsp2.1348)
Supplement: Supplementary file 7 — Data S7. Tabular overview of quality‐of‐life survey outcomes derived from SF36 or SF12 questionnaires. [file JSP2-7-e1348-s010.pdf]

Additional file 7.I Tabular overview of quality-of-life survey outcomes derived from SF36 or SF12 questionnaires.

Author	Ref	Product(s)	Score type	Final FU (Months)	Baseline score	Score at final FU	Score change	Est. change ‡ (% of baseline)
Coric	1	AC	SF36-PCS	12 M	35.3	46.9	11.6	33%
			SF36-MCS	12 M	48.5	50.5	2.0	4%
Zhang	2	Disc allograft	SF36-PCS	84 M	32.3	77.9	45.6	141%
			SF36-MCS	84 M	20.5	75.0	54.5	266%
Xuan *	3	IVD-C	SF36-PCS	72 M	21.4	81.1	59.7	278% *
			SF36-MCS	72 M	26.0	80.8	54.8	211% *
Orozco	4	BM-MSC	SF36-PCS	12 M	12.7	24.8	12.1	95%
			SF36-MCS	12 M	54.1	49.7	-4.4	-8%
Noriega	5-7	BM-MSC	SF12-PCS	12 M	39.0	45.0	6.0	15%
			SF12-MCS	12 M	46.0	48.0	2.0	4%
Xu *	8	BMA	SF36-PCS	24 M	21.8	40.8	19.1	88% *
			SF36-MCS	24 M	20.7	46.2	25.5	123% *
Wolff	9	BMC	SF36	12 M	53.4	68.9	15.5	29%
Monfett	10,11	PRP (unspecified)	SF36-BP	24 M	43.3	67.3	24.0	55%
			SF36-PF	24 M	56.4	74.4	18.0	32%
Zhang	12	LP-PRP	SF36-Pain	12 M	45.0	66.8	21.8	48%
			SF36-PF	12 M	51.8	67.7	15.9	31%
Cheng	13	LR-PRP	SF36-BP	78 M	43.3	80.4	37.1	86%
			SF36-PF	78 M	56.4	82.9	26.5	47%
Ruiz-Lopez	14	LR-PRP	SF36-PCS	6 M	140.1	226.1	86.0	61%
Comella	15	SVF + PRP (unspecified)	SF12-PCS	6 M	29.7	35.1	5.4	18%
			SF12-MCS	6 M	45.6	44.1	-1.5	-3%

* Cell transplantation was performed concurrently with microdiscectomy ‡ Estimated change as percentage from baseline with cell color representing low (red = 0% or less) to high (blue = 100% or more) improvement rates. Abbreviations: AT; adipose tissue – AC: articular chondrocyte – BMA; bone marrow aspirate – BMC; bone marrow concentrate – BM-MSC; bone marrow mesenchymal stromal cell – BP: Bodily pain – FU: Maximal follow-up (in months) – IVD-C; Intervertebral disc cells – LR-PRP: leucocyte rich platelet rich plasma – MCS: Mental component summary – NPC: nucleus pulposus cell – PCS: Physical component summary – PF: Physical functioning – PRP: platelet rich plasma – QoL: Quality-of-life – SF: Short form – SVF: stromal vascular fraction

REFERENCES

1. Coric D, Pettine K, Sumich A, Boltes MO. Prospective study of disc repair with allogeneic chondrocytes presented at the 2012 Joint Spine Section Meeting. *Journal of neurosurgery Spine.* 2013;18(1):85-95.
2. Zhang J, Ruan D, Xuan A, et al. Comparative study of outcomes between allograft intervertebral disc transplantation and anterior cervical discectomy and fusion: a retrospective cohort study at least 5 years of follow-up. *Eur Spine J.* 2023.
3. Xuan A, Ruan D, Wang C, et al. Intradiscal Injection of Autologous Discogenic Cells in Patients with Discectomy: A Prospective Clinical Study of Its Safety and Feasibility. *Stem cells translational medicine.* 2022;11(5):490-503.
4. Orozco L, Soler R, Morera C, Alberca M, Sanchez A, Garcia-Sancho J. Intervertebral disc repair by autologous mesenchymal bone marrow cells: a pilot study. *Transplantation.* 2011;92(7):822-828.
5. Noriega DC, Ardura F, Hernandez-Ramajo R, et al. Intervertebral Disc Repair by Allogeneic Mesenchymal Bone Marrow Cells: A Randomized Controlled Trial. *Transplantation.* 2017;101(8):1945-1951.
6. Garcia-Sancho J, Sanchez A, Vega A, Noriega DC, Nocito M. Influence of HLA Matching on the Efficacy of Allogeneic Mesenchymal Stromal Cell Therapies for Osteoarthritis and Degenerative Disc Disease. *Transplant Direct.* 2017;3(9):e205.
7. Noriega DC, Ardura F, Hernandez-Ramajo R, et al. Treatment of Degenerative Disc Disease With Allogeneic Mesenchymal Stem Cells: Long-term Follow-up Results. *Transplantation.* 2021;105(2):e25-e27.
8. Xu B, Zhang H, Du L, et al. Selective Retention of Bone Marrow Stromal Cells with Gelatin Sponge for Repair of Intervertebral Disc Defects after Microendoscopic Discectomy: A Prospective Controlled Study and 2-Year Follow-Up. *BioMed research international.* 2021;2021:4822383.
9. Wolff M, Shillington JM, Rathbone C, Piasecki SK, Barnes B. Injections of concentrated bone marrow aspirate as treatment for Discogenic pain: a retrospective analysis. *BMC musculoskeletal disorders.* 2020;21(1):135.
10. Tuakli-Wosornu YA, Terry A, Boachie-Adjei K, et al. Lumbar Intradiskal Platelet-Rich Plasma (PRP) Injections: A Prospective, Double-Blind, Randomized Controlled Study. *PM & R : the journal of injury, function, and rehabilitation.* 2016;8(1):1-10; quiz 10.
11. Monfett M, Harrison J, Boachie-Adjei K, Lutz G. Intradiscal platelet-rich plasma (PRP) injections for discogenic low back pain: an update. *Int Orthop.* 2016;40(6):1321-1328.
12. Zhang J, Liu D, Gong Q, Chen J, Wan L. Intradiscal Autologous Platelet-Rich Plasma Injection for Discogenic Low Back Pain: A Clinical Trial. *BioMed research international.* 2022;2022:9563693.
13. Cheng J, Santiago KA, Nguyen JT, Solomon JL, Lutz GE. Treatment of symptomatic degenerative intervertebral discs with autologous platelet-rich plasma: follow-up at 5-9 years. *Regen Med.* 2019;14(9):831-840.
14. Ruiz-Lopez R, Tsai YC. A Randomized Double-Blind Controlled Pilot Study Comparing Leucocyte-Rich Platelet-Rich Plasma and Corticosteroid in Caudal Epidural Injection for Complex Chronic Degenerative Spinal Pain. *Pain practice : the official journal of World Institute of Pain.* 2020;20(6):639-646.
15. Comella K, Silbert R, Parlo M. Effects of the intradiscal implantation of stromal vascular fraction plus platelet rich plasma in patients with degenerative disc disease. *J Transl Med.* 2017;15(1):12.
