# Supplementary material for: A comprehensive review of cell transplantation and platelet‐rich plasma therapy for the treatment of disc degeneration‐related back and neck pain: A systematic evidence‐based analysis
Source: JOR Spine. 2024 Jun 24;7(2):e1348. doi: 10.1002/jsp2.1348 (PMC11196836; doi:10.1002/jsp2.1348)
Supplement: Supplementary file 8 — Data S8. Tabular overview of radiographic reported outcomes. [file JSP2-7-e1348-s003.pdf]

Additional file 8.I Tabular overview of radiographic reported outcomes.

Author	Ref	Product(s)	Score type	Final FU (Months)	Baseline values	Value at final FU	Value change	Est. change ‡ (% of baseline)
Xuan *	1	IVD-C	T2 #	72 M	32.2	25.9	-6.3	-20%
Orozco	2	BM-MSC	T2 †	12 M	0.6	0.7	0.1	16%
Noriega	3-5	BM-MSC	T2 †	12 M	0.5	0.5	0.1	13%
Akeda	6	PL	T2 §	12 M	0.6	0.6	0.0	0%
Ruan	7	Disc allograft	Grey scale	66 M	0.4	0.4	0.0	-7%
Zhang	8	Disc allograft	RVG	84 M	44.8	36.1	-14.7	-33%
Kumar	9	AD-MSC (Low)	ADC	12 M	1278.6	1296.0	17.4	1%
		AD-MSC (High)	ADC	12 M	1064.4	1122.1	57.7	5%
Xuan *	1	IVD-C	Pfirrmann	72 M	2.7	3.2	0.5	-
Mochida	10	NPC	Pfirrmann	12 M	3.0	2.9	-0.1	-
Noriega	3-5	BM-MSC	Pfirrmann	42 M	3.6	2.9	-0.7	-
Lewandrowski	11	UC-MSC	Pfirrmann	24 M	4.1	3.7	0.7	-
Xu *	12	BMA	Pfirrmann	12 M	4.2	4.2	0.0	-
Pettine	13-15	BMC	Mod Pfirrmann	12 M	6.1	5.9	-0.2	-
Kirchner	16	LP-PRP	Pfirrmann	36 M	3.0	3.0	0.0	-
Kirchner	17	LP-PRP	Pfirrmann	6 M	5.0	5.0	0.0	-
Akeda	6	PL	Pfirrmann	12 M	4.0	4.0	0.0	-
Zhang	8	Disc allograft	DHI	84 M	42.9	29.3	-14.7	-34%
Xuan *	1	IVD-C	DHI	72 M	36.8	31.4	-5.4	-15%
Ruan	7	Disc allograft	DH	66 M	5.3	4.3	-1.0	-19%
Xu *	12	BMA	DH	12 M	-	-	-	-18%
El-Kadiry	18	BMC (Intradiscal)	DH	12 M	7.4	8.3	0.8	11%

* Cell transplantation was performed concurrently with microdiscectomy ‡ Estimated change as percentage from baseline with cell color representing low (red = -100%) to high (blue = 100%), # Ratio values of the grayscale normalized to cerebrospinal fluid, † Ratio of T2 values relative to healthy disc, § T2 values normalized to L3/4 disc. Abbreviations: ADC: apparent diffusion coefficient – BMA; bone marrow aspirate – BMC; bone marrow concentrate – BM-MSC; bone marrow mesenchymal stromal cell – FU: Maximal follow-up (in months) – DH: disc height – DHI: dish height index – IVD-C: Intervertebral disc cells – Mod Pfirrmann: Modified Pfirrmann – PL: platelet lysate – LP-PRP: leucocyte poor platelet rich plasma – LR-PRP: leucocyte rich platelet rich plasma

REFERENCES

1. Xuan A, Ruan D, Wang C, et al. Intradiscal Injection of Autologous Discogenic Cells in Patients with Discectomy: A Prospective Clinical Study of Its Safety and Feasibility. *Stem cells translational medicine.* 2022;11(5):490-503.
2. Orozco L, Soler R, Morera C, Alberca M, Sanchez A, Garcia-Sancho J. Intervertebral disc repair by autologous mesenchymal bone marrow cells: a pilot study. *Transplantation.* 2011;92(7):822-828.
3. Noriega DC, Ardura F, Hernandez-Ramajo R, et al. Intervertebral Disc Repair by Allogeneic Mesenchymal Bone Marrow Cells: A Randomized Controlled Trial. *Transplantation.* 2017;101(8):1945-1951.
4. Garcia-Sancho J, Sanchez A, Vega A, Noriega DC, Nocito M. Influence of HLA Matching on the Efficacy of Allogeneic Mesenchymal Stromal Cell Therapies for Osteoarthritis and Degenerative Disc Disease. *Transplant Direct.* 2017;3(9):e205.
5. Noriega DC, Ardura F, Hernandez-Ramajo R, et al. Treatment of Degenerative Disc Disease With Allogeneic Mesenchymal Stem Cells: Long-term Follow-up Results. *Transplantation.* 2021;105(2):e25-e27.
6. Akeda K, Ohishi K, Takegami N, et al. Platelet-Rich Plasma Releasate versus Corticosteroid for the Treatment of Discogenic Low Back Pain: A Double-Blind Randomized Controlled Trial. *J Clin Med.* 2022;11(2).
7. Ruan D, He Q, Ding Y, Hou L, Li J, Luk KD. Intervertebral disc transplantation in the treatment of degenerative spine disease: a preliminary study. *Lancet (London, England).* 2007;369(9566):993-999.
8. Zhang J, Ruan D, Xuan A, et al. Comparative study of outcomes between allograft intervertebral disc transplantation and anterior cervical discectomy and fusion: a retrospective cohort study at least 5 years of follow-up. *Eur Spine J.* 2023.
9. Kumar H, Ha DH, Lee EJ, et al. Safety and tolerability of intradiscal implantation of combined autologous adipose-derived mesenchymal stem cells and hyaluronic acid in patients with chronic discogenic low back pain: 1-year follow-up of a phase I study. *Stem cell research & therapy.* 2017;8(1):262.
10. Mochida J, Sakai D, Nakamura Y, Watanabe T, Yamamoto Y, Kato S. Intervertebral disc repair with activated nucleus pulposus cell transplantation: a three-year, prospective clinical study of its safety. *European cells & materials.* 2015;29:202-212; discussion 212.
11. Lewandrowski KU, Dowling A, Vera JC, Leon JFR, Telfeian AE, Lorio MP. Pain Relief After Allogenic Stem Cell Disc Therapy. *Pain physician.* 2023;26(2):197-206.
12. Xu B, Zhang H, Du L, et al. Selective Retention of Bone Marrow Stromal Cells with Gelatin Sponge for Repair of Intervertebral Disc Defects after Microendoscopic Discectomy: A Prospective Controlled Study and 2-Year Follow-Up. *BioMed research international.* 2021;2021:4822383.
13. Pettine KA, Murphy MB, Suzuki RK, Sand TT. Percutaneous injection of autologous bone marrow concentrate cells significantly reduces lumbar discogenic pain through 12 months. *Stem cells (Dayton, Ohio).* 2015;33(1):146-156.
14. Pettine K, Suzuki R, Sand T, Murphy M. Treatment of discogenic back pain with autologous bone marrow concentrate injection with minimum two year follow-up. *Int Orthop.* 2016;40(1):135-140.
15. Pettine KA, Suzuki RK, Sand TT, Murphy MB. Autologous bone marrow concentrate intradiscal injection for the treatment of degenerative disc disease with three-year follow-up. *Int Orthop.* 2017;41(10):2097-2103.
16. Kirchner F, Anitua E. Minimally Invasive PRGF Treatment for Low Back Pain and Degenerative Disc Disease. In: Anitua E, Cugat R, Sánchez M, eds. *Platelet Rich Plasma in Orthopaedics and Sports Medicine.* Cham: Springer International Publishing; 2018:259-275.
17. Kirchner F, Pinar A, Milani I, Prado R, Padilla S, Anitua E. Vertebral intraosseous plasma rich in growth factor (PRGF-Endoret) infiltrations as a novel strategy for the treatment of degenerative lesions of endplate in lumbar pathology: description of technique and case presentation. *J Orthop Surg Res.* 2020;15(1):72.
18. El-Kadiry AE, Lumbao C, Rafei M, Shammaa R. Autologous BMAC Therapy Improves Spinal Degenerative Joint Disease in Lower Back Pain Patients. *Front Med (Lausanne).* 2021;8:622573.
