# Supplementary material for: A comprehensive review of cell transplantation and platelet‐rich plasma therapy for the treatment of disc degeneration‐related back and neck pain: A systematic evidence‐based analysis
Source: JOR Spine. 2024 Jun 24;7(2):e1348. doi: 10.1002/jsp2.1348 (PMC11196836; doi:10.1002/jsp2.1348)
Supplement: Supplementary file 9 — Data S9. Tabular overview of reported serious adverse events (SAE) following cell and PRP‐transplantation for discogenic pain. [file JSP2-7-e1348-s006.pdf]

Additional file 9.I Tabular overview of reported serious adverse events (SAE) following cell- and PRP-transplantation for discogenic pain.

Author	FU (max)	Product(s)	SAE (n)	Cases (n)	N	%	Description
Coric	1	AC	0	0	15	0%	
Ruan	6	IVD allograft	0	0	5	0%	
Zhang	>6	IVD allograft	0	0	25	0%	
Meisel	2	IVD-C	0	0	12	0%	
Tschugg	<0.5	IVD-C	1	1	12	8%	Disc protrusion
Schwan	NA	IVD-C	10	10	32	31%	Disc re-herniation
Xuan	6	IVD-C	1	1	18	6%	Disc re-herniation
Mochida	3	NPC	0	0	9	0%	
Hunter	1	NPC	6	2	140 (+35)[#]	1%	Myocardial infarction, transient ischemic attack, bacteremia, osteomyelitis, spinal osteoarthritis, pneumonia
Jung	<0.5	AD-MSC	1	1	1	100%*	Pulmonary embolism, chest pain
Piccirilli	1	AD-MSC	0	0	2	0%	
Kumar	1	AD-MSC	0	0	10	0%	
Bates	1	AD-MSC	0	0	9	0%	
Orozco	1	BM-MSC	0	0	10	0%	
Noriega	3.5	BM-MSC	0	0	12	0%	
Papadimitriou	2	BM-MSC	0	0	10	0%	
Amirdelfan	3	MPC	*unclear*	8	60	13%	*unclear*
Lewandrowski	2	UC-MSC	0	0	33	0%	
Pang	2	UC-MSC	0	0	2	0%	
Xu	2	BMA	0	0	15	0%	
Atluri	1	BMC	0	0	40	0%	
Haines	1	BMC	0	0	32	0%	
Pettine	3	BMC	0	0	26	0%	
Wolff	1	BMC	0	0	33	0%	
El-Kadiry	1	BMC	0	0	18	0%	
Jerome	<0.5	BMC	3	3	3	100%*	Spondylodiscitis, epidural abscess
Centeno	2	PL	0	0	470	0%	
Akeda	1	PL	0	0	15	0%	
Akeda	5.9	PL	0	0	11	0%	
Kirchner	0.5	LP-PRP	0	0	86	0%	
Kirchner	0.5	LP-PRP	0	0	1	0%	
Beatty	1	LP-PRP	1	1	1	100%*	Spondylodiscitis
Bise	<0.5	LP-PRP	0	0	30	0%	
Kirchner	2	LP-PRP	0	0	65	0%	
Zielinski	<0.5	LP-PRP	0	0	18	0%	
Zhang	1	LP-PRP	1	1	31	3%	Discitis
Lam	0.8	LP-PRP	0	0	1	0%	
Le	1	LP-PRP or LR-PRP	0	0	25	0%	
Levi	0.5	LR-PRP	0	0	22	0%	
Ruiz-Lopez	0.5	LR-PRP	0	0	25	0%	
Jain	0.5	LR-PRP	0	0	20	0%	
Lam	<0.5	LR-PRP	0	0	3	0%	
Kawabata	0.5	LR-PRP	0	0	2	0%	
Monfett	1	PRP (unspecified)	0	0	29 (+18)[#]	0%	
Bhatia	<0.5	PRP (unspecified)	0	0	10	0%	
Demirci	1.2	PRP (unspecified)	0	0	31	0%	
Navani	0.5	PRP (unspecified)	0	0	6	0%	
Wongjarupong	0.5	PRP (unspecified)	0	0	15	0%	
Saraf	0.5	PRP (unspecified)	0	0	29	0%	
Lutz	1	PRP (unspecified)	0	0	1	0%	
Lam	<0.5	PRP (unspecified)	0	0	1	0%	
Wu	<0.5	PRP (unspecified)	0	0	2	0%	
Xu	1	PRP (unspecified)	0	0	61	0%	
Jiang	1	PRP (unspecified)	1	1	51	2%	Disc re-herniation
Godek	0.5	PRP (unspecified)	0	0	108	0%	
Lutz	3.6	PRP (unspecified)	1	1	37	3%	Spondylodiscitis
Rawson	<0.5	PRP (unspecified)	0	0	2	0%	
Williams	2	PL + PRP and/or PPP	0	0	9	0%	
Subach	1	AT, BMA, plasma	1	1	1	100%*	Discitis, osteomyelitis, epidural abscess, cauda equina syndrome
Centeno	6	BM-MSC + PL	1	1	33	3%	Disc herniation
Ramos	1	BMC + PRP(unspecified)	1	1	1	100%*	Discitis, osteomyelitis
Comella	1	SVF + PRP(unspecified)	0	0	15	0%	
Singh	<0.5	"Stem cells"	1	1	1	100%*	Osteomyelitis, discitis
		Total (Cell)		30	670	4.48%	
		Total (PRP)		7	1286	0.54%	
		Total		34	1906	1.78%	

Additional file to "A Comprehensive Review of Cell Transplantation and Platelet Rich Plasma Therapy for the Treatment of Disc Degeneration-Related Back and Neck Pain: A Systematic Evidence-Based Analysis" by J Schol, S Tamagawa, et al. (2024) JOR Spine

*Selection of patients for this study was performed based on the presence of an adverse event, # Patients from crossover cohort were included in the analysis. Abbreviations: AT; adipose tissue – AC: articular chondrocyte – AD-MSC; adipose derived mesenchymal stromal cells – BMA; bone marrow aspirate –– BMC; bone marrow concentrate – BM-MSC; bone marrow mesenchymal stromal cell – FU: Maximal follow-up (in years) – IVD-C; Intervertebral disc cells – LP-PRP: leucocyte poor platelet rich plasma – LR-PRP: leucocyte rich platelet rich plasma – MPC: mesenchymal precursor cells –NPC: nucleus pulposus cell – PL: platelet lysate – PPP: poor platelet plasma – PRP: platelet rich plasma – SVF: stromal vascular fraction – UC-MSC; umbilical cord mesenchymal stromal cells
