# Supplementary material for: A comprehensive review of cell transplantation and platelet‐rich plasma therapy for the treatment of disc degeneration‐related back and neck pain: A systematic evidence‐based analysis
Source: JOR Spine. 2024 Jun 24;7(2):e1348. doi: 10.1002/jsp2.1348 (PMC11196836; doi:10.1002/jsp2.1348)
Supplement: Supplementary file 10 — Data S10. Tabular overview of patient indications recruited or included into the trials and case reports. [file JSP2-7-e1348-s007.pdf]

Additional file 10.l Tabular overview of patient indications recruited or included into the trials and case reports.

Author	Ref	Product(s)	Country	Discography	Discogenic pain	MRI confirmed	Disc herniation	HIZ	Modic change	Radiculopathy	**Patient indication** *(as defined by authors)*
Coric	1	AC	USA	✓	✓	✓	✗	✗	♦	✗	Chronic (>3M) LBP with single level symptomatic lumbar DDD (L3-S1)
Ruan	2	Disc allograft	China	ns	ns	♦	✓	ns	ns	ns	Patients with cervical spondylotic myelopathy with single-level disc herniation and a patient with traumatic herniation with paraplegia.
Zhang	3	Disc allograft	China	ns	ns	✓	✓	ns	ns	♦	Cervical degenerative disc disease
Meisel	4,5	IVD-C	Germany	ns	ns	ns	ns	ns	♦	ns	Single level DDD indicated for surgical intervention
Tschugg	6	IVD-C	Austria	ns	ns	✓	✓	ns	♦	♦	Single level symptomatic LDH
Schwan	7	IVD-C	Germany	ns	ns	✓	✓	ns	♦	♦	Single level symptomatic LDH
Xuan	8	IVD-C	China	ns	ns	✓	✓	ns	♦	♦	Single level symptomatic LDH
Mochida	9	NPC	Japan	ns	♦	✓	♦	ns	ns	♦	Patients with moderate DDD in adjacent disc scheduled for fusion
Hunter	10-12	NPC	USA	♦	✓	✓	ns	ns	♦	ns	One- or two-level painful mod Pfirrmann grades 3–6 lumbar DDD
Jung *	13	AD-MSC	South Korea	ns	ns	ns	✓	ns	ns	ns	Pulmonary embolism after intravenous AD-MSC for cervical herniated disc *
Piccirilli *	14	AD-MSC	Italy	ns	ns	✓	♦	ns	ns	ns	Patients who have undergone surgery for herniation or segmental spinal stenosis, with evidence of a black disc on MRI*
Kumar	15	AD-MSC	South Korea	✓	✓	✓	ns	ns	♦	ns	Chronic discogenic LBP (>3M) with moderate DDD (Pfirrmann grade III–IV at one or two levels)
Bates	16	AD-MSC	Australia	✓	✓	✓	♦	ns	♦	♦	Chronic (>6M) LBP of discogenic origin
Orozco	17	BM-MSC	Spain	✓	✓	✓	ns	✗	✗	♦	Chronic (>6M) LBP by one or two level DDD with preserved external AF
Noriega	18-20	BM-MSC	Spain	ns	✓	✓	ns	ns	♦	✗	Chronic (>6M) LBP with one or two level DDD (Pfirrmann grade II–IV)
Papadimitriou	21	BM-MSC	Sweden	ns	ns	✓	ns	ns	ns	✗	Chronic (>2Y) LBP with one or two level DDD (Pfirrmann grade III–IV)
Amirdelfan	22,23	MPC	USA	♦	✓	✓	✗	ns	ns	ns	Chronic (>6M) LBP with one level moderate DDD (mod Pfirrmann III－VI)
Lewandrowski	24	UC-MSC	USA	✓	✓	✓	ns	ns	ns	✗	Single level moderate DDD with chronic LBP
Pang	25	UC-MSC	China	✓	✓	✓	✗	ns	ns	✗	Chronic (>2Y) discogenic LBP
Xu	26	BMA	China	ns	ns	✓	✓	ns	ns	✓	LDH with radicular pain (>3M)
Atluri	27	BMC	USA	✗	♦	♦	♦	ns	ns	♦	Chronic (>6M) LBP due to severe lumbar spinal degeneration involving multiple spinal structures
Haines	28	BMC	USA	✓	✓	♦	ns	ns	♦	♦	Discogenic LBP
Pettine	29-31	BMC	USA	♦	✓	✓	✗	ns	♦	✗	Chronic (>6M) moderate to severe discogenic LBP (mod. Pfirrmann IV－VII)
Wolff	32	BMC	USA	✓	✓	✓	ns	✓	♦	♦	Chronic discogenic LBP
El-Kadiry	33	BMC	Canada	✓	✓	✓	ns	ns	ns	ns	Chronic (>6M) discogenic LBP
Jerome *	34	BMC	USA	ns	ns	✓	♦	ns	ns	✓	Spondylodiscitis following intradiscal injection with BMC*
Centeno	35	PL	USA	ns	ns	✓	♦	ns	ns	✓	Patients with lumbar radicular pain
Akeda	36,37	PL	Japan	✓	✓	✓	♦	♦	♦	ns	Chronic (>3M) LBP with one or multiple level(s) MRI confirmed DDD and discography-concordant pain
Akeda	38,39	PL	Japan	✓	✓	✓	ns	ns	ns	✗	Chronic (>3M) discogenic LBP without leg pain
Kirchner	40	LP-PRP	Spain	ns	ns	✓	✓	ns	ns	✓	LBP (≥3M) with symptoms of lumbago and sciatica to right lower feet
Kirchner	41	LP-PRP	Spain	ns	ns	✓	ns	ns	ns	✓	Chronic (>3M) LBP and/or sciatica with DDD
Kirchner	42	LP-PRP	Spain	ns	ns	✓	✓	ns	✓	✓	Chronic (>10Y) LBP with complex disc pathology
Beatty *	43	LP-PRP	USA	✓	✓	✓	ns	ns	ns	ns	Spondylodiscitis following intradiscal PRP injection*
Bise	44	LP-PRP	France	ns	ns	✓	✓	ns	ns	✓	Unilateral resistant lumbar radicular pain related to LDH
Kirchner	45	LP-PRP	Spain	ns	ns	✓	♦	♦	♦	♦	Chronic (>3M) LBP or neck pain
Zielinski	46	LP-PRP	USA	✓	✓	✓	✗	ns	ns	✗	Chronic (>3M) discogenic LBP
Li	47	LP-PRP	China	ns	ns	✓	✓	ns	ns	✓	Single level LDH (L3-L5)
Zhang	48	LP-PRP	China	✓	✓	✓	♦	♦	♦	ns	Chronic (>6M) discography-concordant LBP with MRI positive findings
Lam	49	LP-PRP	China	ns	ns	✓	✓	ns	ns	✓	Radiating neck pain (>3M)
Le	50	LP-PRP or LR-PRP	Vietnam	ns	ns	✓	✓	ns	ns	✓	Chronic (>3M) LBP and unilateral radicular pain with LDH (L4/L5 or L5/S1)
Levi	51	LR-PRP	USA	♦	✓	✓	♦	♦	♦	♦	Discogenic LBP
Cheng	52	LR-PRP	USA	✓	✓	✓	♦	ns	ns	ns	Chronic (>6M) discogenic LBP
Ruiz-Lopez	53	LR-PRP	Spain	ns	ns	✓	ns	ns	ns	♦	Chronic (>3M) LBP with complex degenerative lumbar spinal conditons
Jain	54	LR-PRP	India	✓	✓	✓	ns	♦	♦	♦	Chronic (>6M) discogenic LBP
Lam	55	LR-PRP	China	ns	ns	✓	✗	ns	♦	✗	Chronic (>3Y) LBP
Kawabata	56	LR-PRP	Japan	ns	✓	✓	ns	ns	✓	ns	LBP with Modic 1 change
Monfett	57,58	PRP (unspecified)	USA	✓	✓	✓	♦	ns	ns	ns	Chronic (>6M) discogenic LBP
Bhatia	59	PRP (unspecified)	India	ns	ns	✓	✓	ns	ns	♦	LBP ± radiculopathy (>1M) with lumber disc herniation/prolapse
Demirci	60	PRP (unspecified)	Türkiye	ns	ns	✓	♦	ns	ns	✓	Unrelieved radiculopathy due to “disc pathology”
Navani	61	PRP (unspecified)	USA	✓	✓	✓	♦	ns	ns	✓	Discogenic LBP (>6M) with IVD ≥50% disc height loss, annulus fissures/protrusion, and discography-concordant pain
Karamanakos	62	PRP (unspecified)	Greece	✗	ns	✓	✓	ns	ns	✗	LBP following previous (15Y ago) discectomy
Singh	63	PRP (unspecified)	USA	ns	✓	✓	✓	ns	ns	✓	LBP related to disc prolapse
Wongjarupong	64	PRP (unspecified)	Thailand	ns	ns	✓	✓	ns	ns	✓	Unilateral single level LDH with “correlated clinical” symptoms
Saraf	65	PRP (unspecified)	India	ns	✓	✓	✓	ns	ns	✓	Lumbar radiculopathy secondary to LDH limited to single dermatome
Lutz *	66	PRP (unspecified)	USA	✓	✓	✓	✗	ns	✓	✗	Chronic (>1Y) discogenic LBP*
Lam	67	PRP (unspecified)	China	✓	✓	✓	✗	ns	ns	✓	Chronic neck pain with C5/6 and C6/7 DDD
Wu	68	PRP (unspecified)	Taiwan	ns	✓	✓	✗	✓	ns	✗	Chronic (>3Y) discogenic LBP with LDH
Xu	69	PRP (unspecified)	China	ns	ns	✓	✓	ns	ns	✓	Chronic (>3M) LBP and unilateral radicular pain
Jiang	70	PRP (unspecified)	China	ns	ns	✓	✓	ns	ns	✓	LBP and leg pain (>2M) with LDH

Godek	[71]	PRP (unspecified)	Poland	ns	ns	ns	ns	ns	ns	ns	Lumbar or cervical DDD, or lumbar spinal stenosis
Lutz	[72]	PRP (unspecified)	USA	ns	ns	✓	♦	ns	♦	♦	Chronic (>3M) LBP
Rawson	[73]	PRP + PL	USA	ns	ns	✓	✓	ns	ns	✓	Leg pain with LDH
Williams	[74]	PL with PPP or/and PRP	USA	✗	ns	♦	♦	ns	ns	♦	Axial neck pain
Subach *	[75]	AT, BMA, plasma	USA	ns	ns	✓	ns	ns	ns	✓	Epidural abscess and cauda equina syndrome after intradiscal injection *
Centeno	[76,77]	BM-MSC + PL	USA	ns	ns	✓	✓	ns	ns	✓	LBP and radicular pain with posterior disc bulge
Ramos *	[78]	BMC + PRP	USA	ns	ns	✓	✓	ns	ns	✓	Lumbar Discitis and Osteomyelitis after intradiscal injection *
Comella	[79]	SVF + PRP	USA	ns	ns	✓	ns	ns	♦	✗	Chronic (>6M) LBP with 1-3 level DDD
Singh *	[80]	"Stem cells"	USA	ns	ns	✓	ns	ns	ns	ns	Lumbar spine osteomyelitis and diskitis *

* : Case report/series focused on patient(s) presenting serious adverse events following PRP and/or cell injection, ✓: Defined inclusion criteria, ✗: Defined exclusion criteria, ♦: Mixed inclusion of indication, ns: inclusion of indication not specified

Abbreviations: AC: articular chondrocyte – AD-MSC: adipose derived mesenchymal stromal cells – AF: annulus fibrous – AT: adipose tissue – BMA: bone marrow aspirate – BMC: bone marrow concentrate – BM-MSC: bone marrow mesenchymal stromal cells – DDD: degenerative disc disease – HIZ: high intensity zone – IVD-C: Intervertebral disc cells – LBP: low back pain – LDH: lumbar disc herniation – LP-PRP: leucocyte poor platelet rich plasma – LR-PRP: leucocyte rich platelet rich plasma – MPC: mesenchymal precursor cells – MRI: magnetic resonance imaging – NPC: nucleus pulposus cell – PL: platelet lysate – PPP: platelet poor plasma – PRP: platelet rich plasma – SVF: stromal vascular fraction – UC-MSC: umbilical cord mesenchymal stromal cells

REFERENCE

1. Coric D, Pettine K, Sumich A, Boltes MO. Prospective study of disc repair with allogeneic chondrocytes presented at the 2012 Joint Spine Section Meeting. *Journal of neurosurgery Spine.* 2013;18(1):85-95.
2. Ruan D, He Q, Ding Y, Hou L, Li J, Luk KD. Intervertebral disc transplantation in the treatment of degenerative spine disease: a preliminary study. *Lancet (London, England).* 2007;369(9566):993-999.
3. Zhang J, Ruan D, Xuan A, et al. Comparative study of outcomes between allograft intervertebral disc transplantation and anterior cervical discectomy and fusion: a retrospective cohort study at least 5 years of follow-up. *Eur Spine J.* 2023.
4. Meisel HJ, Ganey T, Hutton WC, Libera J, Minkus Y, Alasevic O. Clinical experience in cell-based therapeutics: intervention and outcome. *Eur Spine J.* 2006;15 Suppl 3(Suppl 3):S397-405.
5. Meisel HJ, Siodla V, Ganey T, Minkus Y, Hutton WC, Alasevic OJ. Clinical experience in cell-based therapeutics: disc chondrocyte transplantation A treatment for degenerated or damaged intervertebral disc. *Biomolecular engineering.* 2007;24(1):5-21.
6. Tschugg A, Diepers M, Simone S, et al. A prospective randomized multicenter phase I/II clinical trial to evaluate safety and efficacy of NOVOCART disk plus autologous disk chondrocyte transplantation in the treatment of nucleotomized and degenerative lumbar disks to avoid secondary disease: safety results of Phase I-a short report. *Neurosurgical review.* 2017;40(1):155-162.
7. Schwan S, Ludtka C, Friedmann A, et al. Calcium Microcrystal Formation in Recurrent Herniation Patients After Autologous Disc Cell Transplantation. *Tissue Eng Regen Med.* 2017;14(6):803-814.
8. Xuan A, Ruan D, Wang C, et al. Intradiscal Injection of Autologous Discogenic Cells in Patients with Discectomy: A Prospective Clinical Study of Its Safety and Feasibility. *Stem cells translational medicine.* 2022;11(5):490-503.
9. Mochida J, Sakai D, Nakamura Y, Watanabe T, Yamamoto Y, Kato S. Intervertebral disc repair with activated nucleus pulposus cell transplantation: a three-year, prospective clinical study of its safety. *European cells & materials.* 2015;29:202-212; discussion 212.
10. Hunter CW, Guyer R, Froimson M, DePalma MJ. Effect of age on outcomes after allogeneic disc tissue supplementation in patients with chronic discogenic low back pain in the VAST trial. *Pain Manag.* 2022;12(3):301-311.
11. Beall DP, Davis T, DePalma MJ, et al. Viable Disc Tissue Allograft Supplementation; One- and Two-level Treatment of Degenerated Intervertebral Discs in Patients with Chronic Discogenic Low Back Pain: One Year Results of the VAST Randomized Controlled Trial. *Pain physician.* 2021;24(6):465-477.
12. Beall DP, Wilson GL, Bishop R, Tally W. VAST Clinical Trial: Safely Supplementing Tissue Lost to Degenerative Disc Disease. *International journal of spine surgery.* 2020;14(2):239-253.
13. Jung JW, Kwon M, Choi JC, et al. Familial occurrence of pulmonary embolism after intravenous, adipose tissue-derived stem cell therapy. *Yonsei medical journal.* 2013;54(5):1293-1296.
14. Piccirilli M, Delfinis CP, Santoro A, Salvati M. Mesenchymal stem cells in lumbar spine surgery: a single institution experience about red bone marrow and fat tissue derived MSCs. *Journal of neurosurgical sciences.* 2017;61(2):124-133.
15. Kumar H, Ha DH, Lee EJ, et al. Safety and tolerability of intradiscal implantation of combined autologous adipose-derived mesenchymal stem cells and hyaluronic acid in patients with chronic discogenic low back pain: 1-year follow-up of a phase I study. *Stem cell research & therapy.* 2017;8(1):262.
16. Bates D, Vivian D, Freitag J, et al. Low-dose mesenchymal stem cell therapy for discogenic pain: safety and efficacy results from a 1-year feasibility study. *Future Sci OA.* 2022;8(5):FSO794.
17. Orozco L, Soler R, Morera C, Alberca M, Sanchez A, Garcia-Sancho J. Intervertebral disc repair by autologous mesenchymal bone marrow cells: a pilot study. *Transplantation.* 2011;92(7):822-828.
18. Garcia-Sancho J, Sanchez A, Vega A, Noriega DC, Nocito M. Influence of HLA Matching on the Efficacy of Allogeneic Mesenchymal Stromal Cell Therapies for Osteoarthritis and Degenerative Disc Disease. *Transplant Direct.* 2017;3(9):e205.
19. Noriega DC, Ardura F, Hernandez-Ramajo R, et al. Treatment of Degenerative Disc Disease With Allogeneic Mesenchymal Stem Cells: Long-term Follow-up Results. *Transplantation.* 2021;105(2):e25-e27.
20. Noriega DC, Ardura F, Hernandez-Ramajo R, et al. Intervertebral Disc Repair by Allogeneic Mesenchymal Bone Marrow Cells: A Randomized Controlled Trial. *Transplantation.* 2017;101(8):1945-1951.
21. Papadimitriou N, Hebelka H, Hingert D, et al. Intradiscal Injection of Iron-Labeled Autologous Mesenchymal Stromal Cells in Patients With Chronic Low Back Pain: A Feasibility Study With 2 Years Follow-Up. *International journal of spine surgery.* 2021;15(6):1201-1209.
22. Amirdelfan K, Bae H, McJunkin T, et al. Allogeneic mesenchymal precursor cells treatment for chronic low back pain associated with degenerative disc disease: a prospective randomized, placebo-controlled 36-month study of safety and efficacy. *The spine journal : official journal of the North American Spine Society.* 2021;21(2):212-230.
23. Ju DG, Kanim LE, Bae HW. Is There Clinical Improvement Associated With Intradiscal Therapies? A Comparison Across Randomized Controlled Studies. *Global spine journal.* 2022;12(5):756-764.
24. Lewandrowski KU, Dowling A, Vera JC, Leon JFR, Telfeian AE, Lorio MP. Pain Relief After Allogenic Stem Cell Disc Therapy. *Pain physician.* 2023;26(2):197-206.
25. Pang X, Yang H, Peng B. Human umbilical cord mesenchymal stem cell transplantation for the treatment of chronic discogenic low back pain. *Pain physician.* 2014;17(4):E525-530.
26. Xu B, Zhang H, Du L, et al. Selective Retention of Bone Marrow Stromal Cells with Gelatin Sponge for Repair of Intervertebral Disc Defects after Microendoscopic Discectomy: A Prospective Controlled Study and 2-Year Follow-Up. *BioMed research international.* 2021;2021:4822383.
27. Atluri S, Murphy MB, Dragella R, et al. Evaluation of the Effectiveness of Autologous Bone Marrow Mesenchymal Stem Cells in the Treatment of Chronic Low Back Pain Due to Severe Lumbar Spinal Degeneration: A 12-Month, Open-Label, Prospective Controlled Trial. *Pain physician.* 2022;25(2):193-207.
28. Haines CM, Bhatt FR, Orosz LD, et al. Low Back Pain, Disability, and Quality of Life One Year following Intradiscal Injection of Autologous Bone Marrow Aspirate Concentrate. *Stem Cells Int.* 2022;2022:9617511.
29. Pettine K, Suzuki R, Sand T, Murphy M. Treatment of discogenic back pain with autologous bone marrow concentrate injection with minimum two year follow-up. *Int Orthop.* 2016;40(1):135-140.
30. Pettine KA, Murphy MB, Suzuki RK, Sand TT. Percutaneous injection of autologous bone marrow concentrate cells significantly reduces lumbar discogenic pain through 12 months. *Stem cells (Dayton, Ohio).* 2015;33(1):146-156.
31. Pettine KA, Suzuki RK, Sand TT, Murphy MB. Autologous bone marrow concentrate intradiscal injection for the treatment of degenerative disc disease with three-year follow-up. *Int Orthop.* 2017;41(10):2097-2103.
32. Wolff M, Shillington JM, Rathbone C, Piasecki SK, Barnes B. Injections of concentrated bone marrow aspirate as treatment for Discogenic pain: a retrospective analysis. *BMC musculoskeletal disorders.* 2020;21(1):135.
33. El-Kadiry AE, Lumbao C, Rafei M, Shammaa R. Autologous BMAC Therapy Improves Spinal Degenerative Joint Disease in Lower Back Pain Patients. *Front Med (Lausanne).* 2021;8:622573.
34. Jerome MA, Lutz C, Lutz GE. Risks of Intradiscal Orthobiologic Injections: A Review of the Literature and Case Series Presentation. *International journal of spine surgery.* 2021;15(s1):26-39.

35. Centeno C, Markle J, Dodson E, et al. The use of lumbar epidural injection of platelet lysate for treatment of radicular pain. *J Exp Orthop.* 2017;4(1):38.
36. Akeda K, Fujiwara T, Takegami N, Yamada J, Sudo A. Retrospective Analysis of Factors Associated with the Treatment Outcomes of Intradiscal Platelet-Rich Plasma-Releasate Injection Therapy for Patients with Discogenic Low Back Pain. *Medicina (Kaunas).* 2023;59(4).
37. Akeda K, Ohishi K, Takegami N, et al. Platelet-Rich Plasma Releasate versus Corticosteroid for the Treatment of Discogenic Low Back Pain: A Double-Blind Randomized Controlled Trial. *J Clin Med.* 2022;11(2).
38. Akeda K, Ohishi K, Masuda K, et al. Intradiscal Injection of Autologous Platelet-Rich Plasma Releasate to Treat Discogenic Low Back Pain: A Preliminary Clinical Trial. *Asian Spine J.* 2017;11(3):380-389.
39. Akeda K, Takegami N, Yamada J, et al. Platelet-Rich Plasma-Releasate (PRPr) for the Treatment of Discogenic Low Back Pain Patients: Long-Term Follow-Up Survey. *Medicina (Kaunas).* 2022;58(3).
40. Kirchner F, Anitua E. Minimally Invasive PRGF Treatment for Low Back Pain and Degenerative Disc Disease. In: Anitua E, Cugat R, Sánchez M, eds. *Platelet Rich Plasma in Orthopaedics and Sports Medicine.* Cham: Springer International Publishing; 2018:259-275.
41. Kirchner F, Anitua E. Intradiscal and intra-articular facet infiltrations with plasma rich in growth factors reduce pain in patients with chronic low back pain. *J Craniovertebr Junction Spine.* 2016;7(4):250-256.
42. Kirchner F, Pinar A, Milani I, Prado R, Padilla S, Anitua E. Vertebral intraosseous plasma rich in growth factor (PRGF-Endoret) infiltrations as a novel strategy for the treatment of degenerative lesions of endplate in lumbar pathology: description of technique and case presentation. *J Orthop Surg Res.* 2020;15(1):72.
43. Beatty NR, Lutz C, Boachie-Adjei K, Leynes TA, Lutz C, Lutz G. Spondylodiscitis due to Cutibacterium acnes following lumbosacral intradiscal biologic therapy: a case report. *Regen Med.* 2019;14(9):823-829.
44. Bise S, Dallaudiere B, Pesquer L, et al. Comparison of interlaminar CT-guided epidural platelet-rich plasma versus steroid injection in patients with lumbar radicular pain. *Eur Radiol.* 2020;30(6):3152-3160.
45. Kirchner F, Milani I, Martinez A, et al. Plasma Rich in Growth Factors (PRGF) in the Treatment of Cervical and Lumbar Back Pain: A Retrospective Observational Clinical Study. *Pain physician.* 2021;24(5):E649-E660.
46. Zielinski MA, Evans NE, Bae H, et al. Safety and Efficacy of Platelet Rich Plasma for Treatment of Lumbar Discogenic Pain: A Prospective, Multicenter, Randomized, Double-blind Study. *Pain physician.* 2022;25(1):29-34.
47. Li J, Yuan X, Li F, et al. A novel full endoscopic annular repair technique combined with autologous conditioned plasma intradiscal injection: a new safe serial therapeutic model for the treatment of lumbar disc herniation. *Ann Palliat Med.* 2021;10(1):292-301.
48. Zhang J, Liu D, Gong Q, Chen J, Wan L. Intradiscal Autologous Platelet-Rich Plasma Injection for Discogenic Low Back Pain: A Clinical Trial. *BioMed research international.* 2022;2022:9563693.
49. Lam KHS, Hung CY, Wu TJ, et al. Novel Ultrasound-Guided Cervical Intervertebral Disc Injection of Platelet-Rich Plasma for Cervicodiscogenic Pain: A Case Report and Technical Note. *Healthcare (Basel).* 2022;10(8).
50. Le VT, Nguyen Dao LT, Nguyen AM. Transforaminal injection of autologous platelet-rich plasma for lumbar disc herniation: A single-center prospective study in Vietnam. *Asian J Surg.* 2023;46(1):438-443.
51. Levi D, Horn S, Tyszko S, Levin J, Hecht-Leavitt C, Walko E. Intradiscal Platelet-Rich Plasma Injection for Chronic Discogenic Low Back Pain: Preliminary Results from a Prospective Trial. *Pain medicine (Malden, Mass).* 2016;17(6):1010-1022.
52. Cheng J, Santiago KA, Nguyen JT, Solomon JL, Lutz GE. Treatment of symptomatic degenerative intervertebral discs with autologous platelet-rich plasma: follow-up at 5-9 years. *Regen Med.* 2019;14(9):831-840.
53. Ruiz-Lopez R, Tsai YC. A Randomized Double-Blind Controlled Pilot Study Comparing Leucocyte-Rich Platelet-Rich Plasma and Corticosteroid in Caudal Epidural Injection for Complex Chronic Degenerative Spinal Pain. *Pain practice : the official journal of World Institute of Pain.* 2020;20(6):639-646.
54. Jain D, Goyal T, Verma N, Paswan AK, Dubey RK. Intradiscal Platelet-Rich Plasma Injection for Discogenic Low Back Pain and Correlation with Platelet Concentration: A Prospective Clinical Trial. *Pain medicine (Malden, Mass).* 2020;21(11):2719-2725.
55. Lam KHS, Hung CY, Wu TJ. Ultrasound-guided L5/S1 intradiscal needle placement using biplanar approach with the patient in the lateral decubitus position: A report of three cases. *Pain practice : the official journal of World Institute of Pain.* 2022;22(1):117-122.
56. Kawabata S, Hachiya K, Nagai S, et al. Autologous Platelet-Rich Plasma Administration on the Intervertebral Disc in Low Back Pain Patients with Modic Type 1 Change: Report of Two Cases. *Medicina (Kaunas).* 2023;59(1).
57. Monfett M, Harrison J, Boachie-Adjei K, Lutz G. Intradiscal platelet-rich plasma (PRP) injections for discogenic low back pain: an update. *Int Orthop.* 2016;40(6):1321-1328.
58. Tuakli-Wosornu YA, Terry A, Boachie-Adjei K, et al. Lumbar Intradiskal Platelet-Rich Plasma (PRP) Injections: A Prospective, Double-Blind, Randomized Controlled Study. *PM & R : the journal of injury, function, and rehabilitation.* 2016;8(1):1-10; quiz 10.
59. Bhatia R, Chopra G. Efficacy of Platelet Rich Plasma via Lumbar Epidural Route in Chronic Prolapsed Intervertebral Disc Patients-A Pilot Study. *J Clin Diagn Res.* 2016;10(9):UC05-UC07.
60. Demirci AY. The retrospective analysis of platelet-rich plasma and corticosteroid injection under epiduroscopic guidance for radiculopathy in operated or unoperated patients for lumbar disc herniation. *Turk J Phys Med Rehabil.* 2022;68(3):409-417.
61. Navani A, Hames A. Platelet-rich plasma injections for lumbar discogenic pain: A preliminary assessment of structural and functional changes. *Techniques in Regional Anesthesia and Pain Management.* 2015;19(1-2):38-44.
62. Karamanakos PN, Manousakis E, Rozakis D, Kamarainen OP, Oikonomi E, Panteli ES. Intradiscal Platelet-Rich Plasma for Discogenic Low Back Pain Owing to a Degenerated and Previously Discectomized L5-S1 Disc. *Pain medicine (Malden, Mass).* 2021;22(5):1235-1236.
63. Singh GK, Talawar P, Kumar A, Sharma RS, Purohit G, Bhandari B. Effect of autologous platelet-rich plasma (PRP) on low back pain in patients with prolapsed intervertebral disc: A randomised controlled trial. *Indian J Anaesth.* 2023;67(3):277-282.
64. Wongjarupong A, Pairuchvej S, Laohapornsvan P, et al. "Platelet-Rich Plasma" epidural injection an emerging strategy in lumbar disc herniation: a Randomized Controlled Trial. *BMC musculoskeletal disorders.* 2023;24(1):335.
65. Saraf A, Hussain A, Sandhu AS, Bishnoi S, Arora V. Transforaminal Injections of Platelet-Rich Plasma Compared with Steroid in Lumbar radiculopathy: A Prospective, Double-Blind Randomized Study. *Indian J Orthop.* 2023;57(7):1126-1133.
66. Lutz GE. Increased Nuclear T2 Signal Intensity and Improved Function and Pain in a Patient One Year After an Intradiscal Platelet-Rich Plasma Injection. *Pain medicine (Malden, Mass).* 2017;18(6):1197-1199.
67. Lam KHS, Hung CY, Wu TJ. Ultrasound-Guided Cervical Intradiscal Injection with Platelet-Rich Plasma with Fluoroscopic Validation for the Treatment of Cervical Discogenic Pain: A Case Presentation and Technical Illustration. *Journal of pain research.* 2020;13:2125-2129.
68. Wu TJ, Hung CY, Lee CW, Lam S, Clark TB, Chang KV. Ultrasound-Guided Lumbar Intradiscal Injection for Discogenic Pain: Technical Innovation and Presentation of Two Cases. *Journal of pain research.* 2020;13:1103-1107.
69. Xu Z, Wu S, Li X, Liu C, Fan S, Ma C. Ultrasound-Guided Transforaminal Injections of Platelet-Rich Plasma Compared with Steroid in Lumbar Disc Herniation: A Prospective, Randomized, Controlled Study. *Neural Plast.* 2021;2021:5558138.
70. Jiang Y, Zuo R, Yuan S, et al. Transforaminal Endoscopic Lumbar Discectomy with versus without Platelet-Rich Plasma Injection for Lumbar Disc Herniation: A Prospective Cohort Study. *Pain Res Manag.* 2022;2022:6181478.
71. Godek P. High Volume PRP Therapy. *Ortop Traumatol Rehabil.* 2022;24(1):43-60.

72. Lutz C, Cheng J, Prysak M, Zukofsky T, Rothman R, Lutz G. Clinical outcomes following intradiscal injections of higher-concentration platelet-rich plasma in patients with chronic lumbar discogenic pain. *Int Orthop.* 2022;46(6):1381-1385.
73. Rawson B. Platelet-Rich Plasma and Epidural Platelet Lysate: Novel Treatment for Lumbar Disk Herniation. *J Am Osteopath Assoc.* 2020;120(3):201-207.
74. Williams C, Jerome M, Fausel C, Dodson E, Stemper I, Centeno C. Regenerative Injection Treatments Utilizing Platelet Products and Prolotherapy for Cervical Spine Pain: A Functional Spinal Unit Approach. *Cureus.* 2021;13(10):e18608.
75. Subach BR, Copay AG, Martin MM, Schuler TC, DeWolfe DS. Epidural abscess and cauda equina syndrome after percutaneous intradiscal therapy in degenerative lumbar disc disease. *The spine journal : official journal of the North American Spine Society.* 2012;12(11):e1-4.
76. Centeno C, Markle J, Dodson E, et al. Treatment of lumbar degenerative disc disease-associated radicular pain with culture-expanded autologous mesenchymal stem cells: a pilot study on safety and efficacy. *J Transl Med.* 2017;15(1):197.
77. Elabd C, Centeno CJ, Schultz JR, Lutz G, Ichim T, Silva FJ. Intra-discal injection of autologous, hypoxic cultured bone marrow-derived mesenchymal stem cells in five patients with chronic lower back pain: a long-term safety and feasibility study. *J Transl Med.* 2016;14(1):253.
78. Ramos O, Speirs JN, Danisa O. Lumbar Discitis and Osteomyelitis After a Spinal Stem Cell Injection?: A Case Report and Literature Review. *JBJS Case Connect.* 2020;10(3):e1900636.
79. Comella K, Silbert R, Parlo M. Effects of the intradiscal implantation of stromal vascular fraction plus platelet rich plasma in patients with degenerative disc disease. *J Transl Med.* 2017;15(1):12.
80. Singh V, McGuffin S, Sabo M, Nicholls M. Lumbar Spine Osteomyelitis and Diskitis following Intradiskal Stem Cell Injections. *PM & R : the journal of injury, function, and rehabilitation.* 2020;12(6):624-625.
